# Sca-1^+^ Cardiosphere-Derived Cells Are Enriched for Isl1-Expressing Cardiac Precursors and Improve Cardiac Function after Myocardial Injury

**DOI:** 10.1371/journal.pone.0030329

**Published:** 2012-01-17

**Authors:** Jianqin Ye, Andrew Boyle, Henry Shih, Richard E. Sievers, Yan Zhang, Megha Prasad, Hua Su, Yan Zhou, William Grossman, Harold S. Bernstein, Yerem Yeghiazarians

**Affiliations:** 1 Division of Cardiology, Department of Medicine, University of California San Francisco, San Francisco, California, United States of America; 2 Cardiovascular Research Institute, University of California San Francisco, San Francisco, California, United States of America; 3 Eli and Edythe Broad Center of Regeneration Medicine and Stem Cell Research, University of California San Francisco, San Francisco, California, United States of America; 4 Department of Pediatrics, University of California San Francisco, San Francisco, California, United States of America; 5 Department of Anesthesia and Perioperative Care, University of California San Francisco, San Francisco, California, United States of America; 6 Department of Cell and Tissue Biology, University of California San Francisco, San Francisco, California, United States of America; Brigham & Women's Hospital - Harvard Medical School, United States of America

## Abstract

**Background:**

Endogenous cardiac progenitor cells are a promising option for cell-therapy for myocardial infarction (MI). However, obtaining adequate numbers of cardiac progenitors after MI remains a challenge. Cardiospheres (CSs) have been proposed to have cardiac regenerative properties; however, their cellular composition and how they may be influenced by the tissue milieu remains unclear.

**Methodology/Principal Finding:**

Using “middle aged” mice as CSs donors, we found that acute MI induced a dramatic increase in the number of CSs in a mouse model of MI, and this increase was attenuated back to baseline over time. We also observed that CSs from post-MI hearts engrafted in ischemic myocardium induced angiogenesis and restored cardiac function. To determine the role of Sca-1^+^CD45^-^ cells within CSs, we cloned these from single cell isolates. Expression of Islet-1 (Isl1) in Sca-1^+^CD45^-^ cells from CSs was 3-fold higher than in whole CSs. Cloned Sca-1^+^CD45^-^ cells had the ability to differentiate into cardiomyocytes, endothelial cells and smooth muscle cells *in vitro*. We also observed that cloned cells engrafted in ischemic myocardium induced angiogenesis, differentiated into endothelial and smooth muscle cells and improved cardiac function in post-MI hearts.

**Conclusions/Significance:**

These studies demonstrate that cloned Sca-1^+^CD45^-^ cells derived from CSs from infarcted “middle aged” hearts are enriched for second heart field (i.e., Isl-1^+^) precursors that give rise to both myocardial and vascular tissues, and may be an appropriate source of progenitor cells for autologous cell-therapy post-MI.

## Introduction

Growing evidence demonstrates the existence of endogenous cardiac progenitor cells in the adult mammalian heart, which can divide and differentiate into cardiomyocytes, endothelial cells and smooth muscle cells, and potentially play an important role in maintaining normal cardiac homeostasis [1] as well as myocardial response to injury [2]–[6]. Various methods have been used to isolate cardiac progenitor cells, including immune selection of cells using various cell surface markers [2]–[4] or *in vitro* culture of cardiospheres (CSs) [7]–[9]. Endogenous cardiac progenitor cells could be collected from the hearts of patients by myocardial biopsy, expanded *in vitro* [8], and then potentially be transplanted back to the same patient to repair damaged myocardium. This approach would avoid immune rejection and may therefore represent an ideal model for cell therapy to achieve long term reconstitution of lost myocardium and preservation of cardiac function [10]–[12]. However, the myocardiogenic potential of CSs and adult cardiac progenitor cells has recently been questioned [13], [14]. In fact, a recent report by Andersen et al suggested that CSs are merely fibroblasts and, therefore, not a potential source of therapeutic cardiac progenitor cells [13]. In addition, whether CSs obtained from age-appropriate tissues have the ability to function in myocardial rehabilitation has not been studied.

During fetal development, the LIM homeodomain transcription factor Islet-1 (Isl1) is expressed in a cell population that gives rise to second heart field structures and the myocardial vasculature, and is accepted as a marker of endogenous cardiac progenitors [5], [6], [15]. The existence of these cells in the adult heart is not clear [6]. Since Isl1 is expressed in the nucleus, it has been difficult to isolate and purify genetically unmodified endogenous Isl1^+^ cells for therapeutic evaluation. Whereas cells bearing the surface markers c-kit and Sca-1 have been isolated from the adult heart and recognized as adult resident cardiac progenitor cells [2]–[4].

Questions remain regarding the behavior and cellular composition of CSs and their response to signals from the myocardial tissue environment, including: 1) whether acute myocardial infarction (MI) effects the generation of CSs; 2) whether CSs derived from injured myocardium have therapeutic potential to repair ischemically damaged hearts *in vivo*; and 3) whether specific subpopulations of CS cells bear the therapeutic potential of CSs *in vivo*. We now demonstrate that the Sca-1^+^CD45^−^ CS subpopulation from post-MI “middle-aged” hearts are enriched in Isl1^+^ cells, have the potential to differentiate into both cardiomyocytes and vascular cells, and can be used to improve cardiac function in the injured heart.

## Results

### CSs enrichment with injury

To determine whether myocardial injury influences the generation of CS-forming cells, whole hearts including the infarct area were removed from mice following experimental MI, as well as from sham-operated and non-operated mice, and were cut to small pieces as “explants”. A monolayer of fibroblast-like cells migrated out from the cardiac explants over several weeks in culture. From this monolayer, small, round, phase-bright cells (CS-forming cells) were seen to emerge (Figure S1A, B). CS-forming cells from non-operated hearts contained several populations of cells, based on their expression of Sca-1, c-kit, CD45, CD133, CD34, Flk1 and CD31 (Figure S2A, B). We observed that explants took less time to form confluent monolayer in culture when isolated from injured hearts (14±1, 13±1 and 18±2 days from 1-, 2- and 4-weeks post-MI hearts, respectively), compared to explants derived from sham-operated and non-operated hearts (21±1 and 32±2 days, respectively). Thus, cells derived from 1- and 2-week post-MI cardiac explants expanded more rapidly than both control groups (P<0.004), however, the growth rate of these cells was attenuated by 4-weeks post-MI and was not significantly different from the sham-operated hearts (P>0.05).

**Figure 1:**
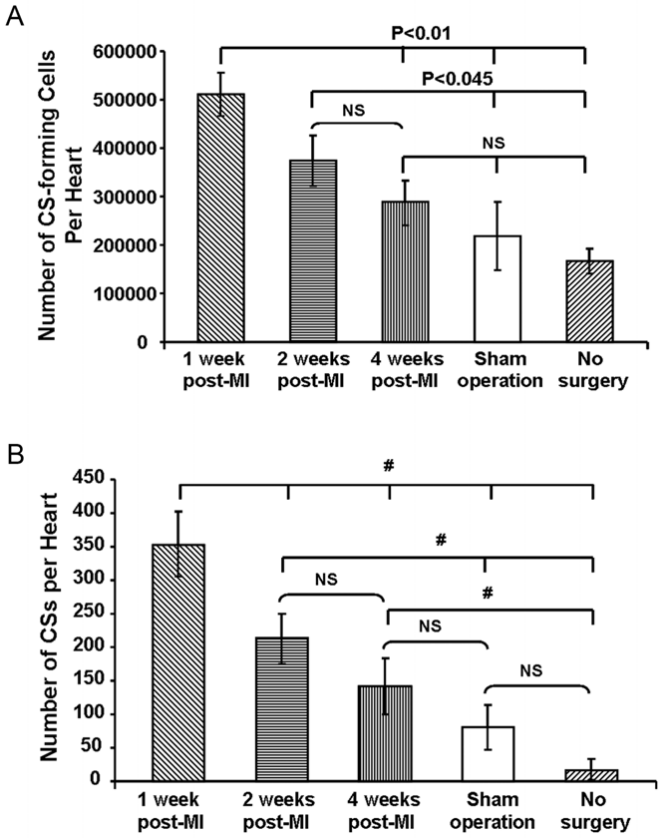
Myocardial injury increases the production of CSs. (A) Numbers of CS-forming cells harvested from hearts at 1-week and 2-weeks post-MI was significantly higher than those from sham-operated and non-operated hearts (N = 6). (B) Hearts at 1- and 2-weeks post-MI generated more CSs than sham-operated and non-operated hearts. # P<0.03. Hearts at 4-weeks post-MI produced similar number of CSs to sham-operated hearts (N = 6). Data in Figure shown as mean±SEM.

In addition to faster growth rates, the number of putative CS-forming cells harvested from hearts at 1-week (5.12×10^5^±0.45×10^5^/heart) and 2-weeks (3.75×10^5^±0.52×10^5^/heart) post-MI was significantly higher than those from sham-operated (2.20×10^5^±0.70×10^5^/heart) and non-operated hearts (1.67×10^5^±0.26×10^5^ cells/heart) (P<0.045) (Figure 1A). MI therefore produced more than twice as many CS-forming cells in approximately half the culture time, equivalent to an approximate four-fold increase in proliferative rate. However, the number of putative CS-forming cells harvested 4-weeks post-MI (2.88×10^5^±0.46×10^5^ cells/heart) hearts was not significantly different from sham-operated and non-operated hearts (P>0.05), suggesting that the increase in the number of CS-forming cells was also attenuated by 4-weeks post-MI (Figure 1A).

Consistent with our observation of an increase in CS-forming cell number with injury, we observed that the number of CSs derived from hearts harvested 1-week (354±50/heart) and 2-weeks (213±38/heart) post-MI were significantly higher than from sham-operated hearts (80±33/heart) (p<0.001) and non-operated hearts (18±14/heart) (P<0.01) (Figure 1B). Our results also showed that the number of CSs from hearts 4-weeks post-MI (141±42/heart) was not significantly different compared to sham operated hearts (P>0.05), but still higher than from non-operated hearts (P = 0.02) (Figure 1B). Thus, the effect of injury on CS formation was attenuated by 4-weeks post-MI.

We have demonstrated that at each of the three stages of CS formation (explant outgrowth, CS-forming cell generation and number of CSs), the proliferative was greater at 1–2 weeks post-MI, and this then returned toward baseline by 4 weeks post-MI.

### No regional differences in CS-yield from infarcted hearts

To determine the CS generating potential of different regions of the heart, we separated the hearts 1-week post-MI into five regions: left ventricle (LV) excluding scar, right ventricle (RV), septum, left atrium (LA) and right atrium (RA), and cultured them separately. The number of CSs from each region of the heart was counted and adjusted for tissue weight. We observed no statistically significant regional differences in CS production (LV: 2.6±0.6 CSs/mg; RV: 3.8±1.0; septum: 2.3±0.5; LA: 2.2±0.2; RA: 2.9±0.7; P>0.05) (Figure S3).

### CSs contain Isl1^+^ cells

**Figure 2:**
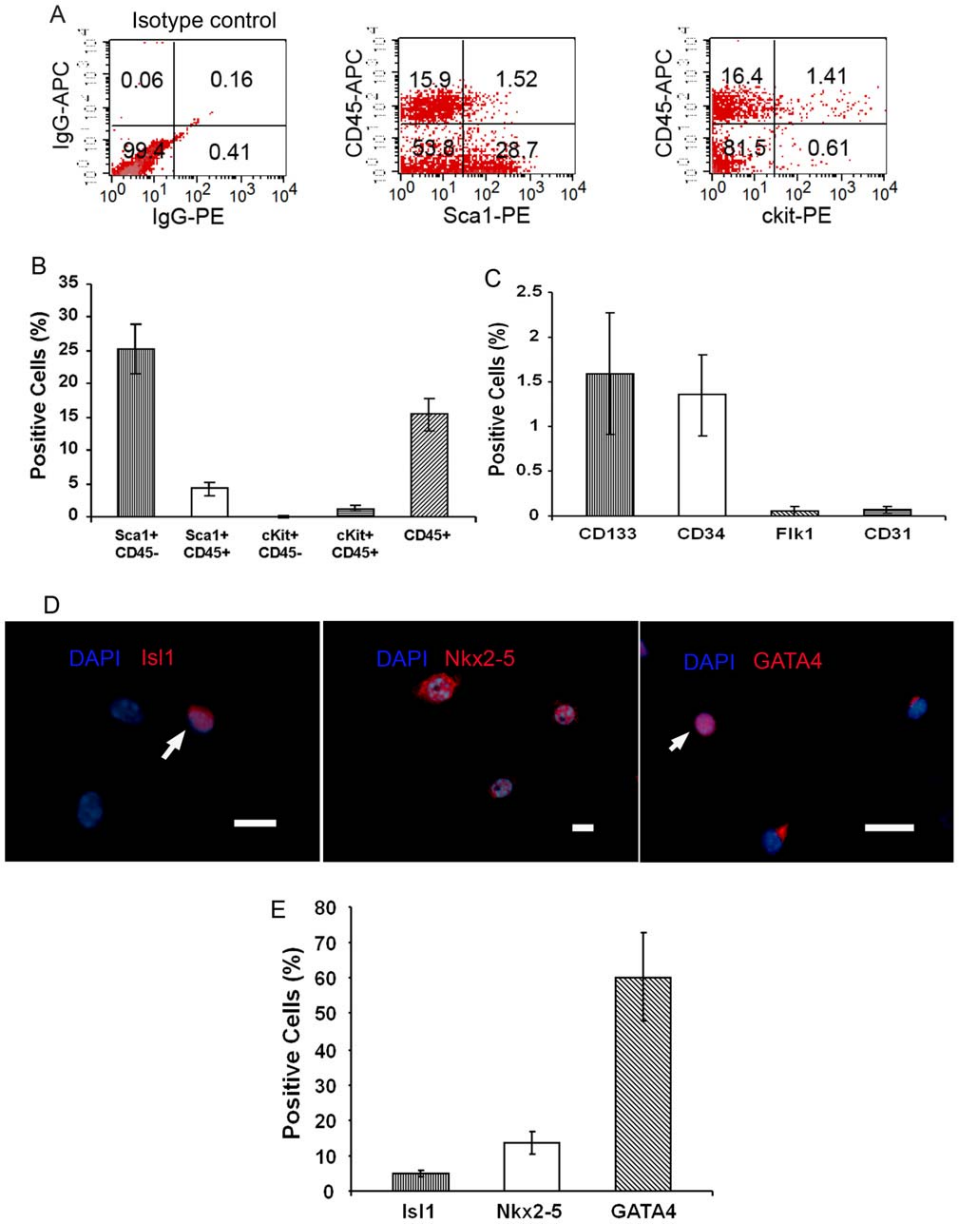
Cellular composition of CSs from mouse hearts. (A) Flow cytometric analysis of Sca-1, CD45 and c-kit expression in disaggregated CS cells. Typical results are shown (N = 6). (B, C) Bar graph showing the profile of progenitor cell markers in CSs by FACS (N = 6). (D) Immunocytochemical staining demonstrates that CS cells express Isl1, Nkx2-5 and GATA4. Arrows point to positive staining cells (red). Nuclei stained with DAPI. Scale bar = 35 µm and 15 µm (NKx2-5). (E) Bar graph shows the profile of Isl1, Nkx2-5, GATA4 positive cells in CSs by immunocytochemical staining. Data in Figure shown as mean±SEM.

**Figure 3:**
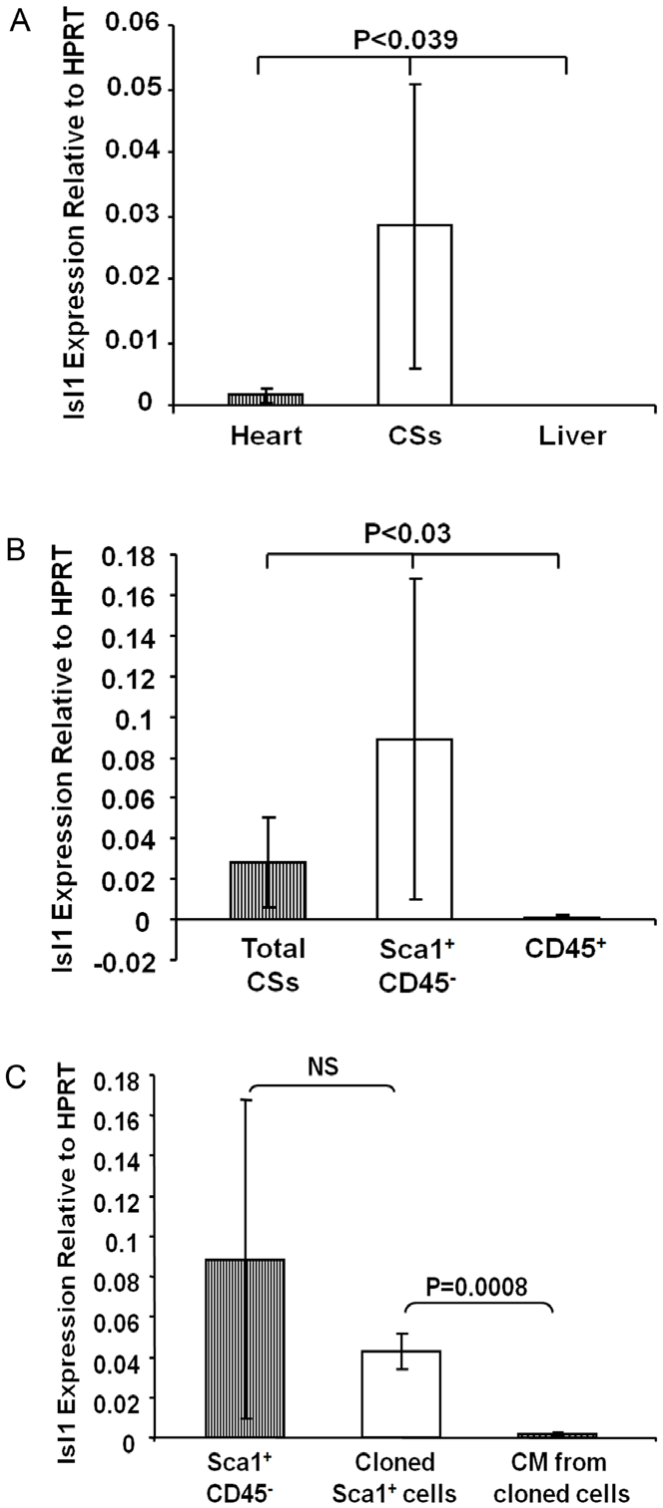
Isl1^+^ cells are present in adult heart and CSs. Real-time RT-PCR was used to compare Isl1 expression in heart, indicated cells, and liver (negative control). Results show as Isl1 mRNA expression relative to Hypoxanthine phosphoribosyltransferase (HPRT). (A) Isl1 expression in CSs (N = 10) was 17-fold higher than in the hearts (n = 4). (B) Isl1 expression in Sca-1^+^CD45^−^ cells (N = 6) derived from CSs was 3-fold higher compared to that in whole CSs (N = 10). (C) Isl1 expression in cloned Sca-1^+^ CD45^−^ cells (n = 5) was similar to primary Sca-1^+^CD45^−^ cells isolated from CSs (N = 6). Isl1 expression dropped significantly with differentiation (N = 3). CM: cardiomyocytes.

**Figure 4:**
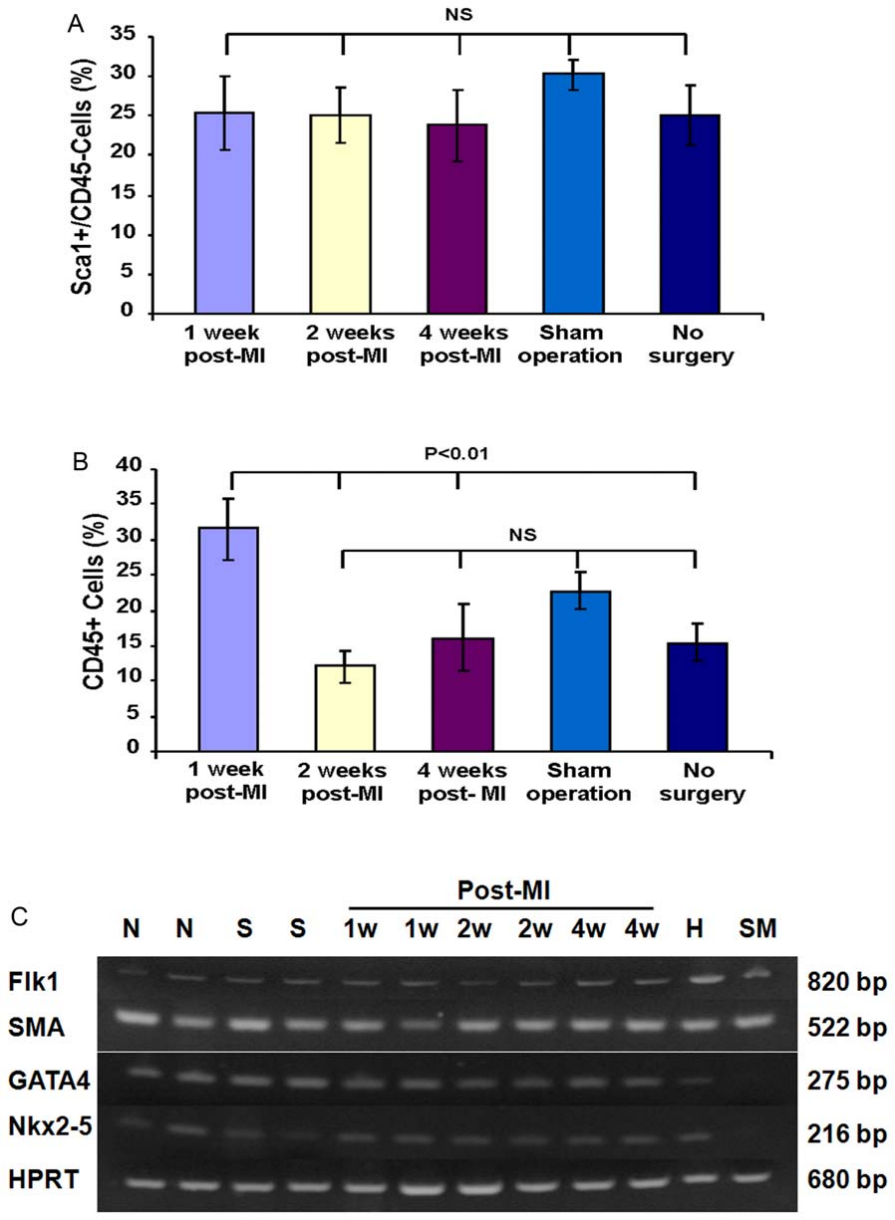
Cellular composition of CSs from injured hearts. (A) The percentages of Sca-1^+^CD45^−^ in CSs were not altered by MI (N = 6). (B) The percentages of total CD45^+^cells in CSs was increased at 1-week post-MI and attenuated at 2-week post-MI (N = 6). (C) Semi-quantitative RT-PCR analysis showed that CSs from all groups expressed similar level of Nkx2-5, GATA4, Flk-1 and SMA. HPRT was used as control. N: Non-surgery. S: Sham-operated. 1W: 1 week post-MI. 2W: 2 weeks post-MI. 4W: 4 weeks post-MI. H: mouse heart (positive control). SM: skeletal muscle (negative control). Data in Figure shown as mean±SEM.

We used fluorescence-activated cell sorting (FACS) to determine the cellular composition of CSs derived from control and infarcted hearts after 14 days in culture. CSs from non-operated hearts contained several populations of cells, based on their expression of Sca-1, c-kit, CD45, CD133, CD34, Flk1 and CD31 (Figure 2A, B and C). Immunocytochemical staining showed that the cardiac transcription factors, Isl1, Nkx2-5 and GATA4, were expressed in 5.0±1.0%, 13.6±1.4%, and 60.1±5.6% of CS cells, respectively (Figure 2D and E). These were confirmed by real-time RT-PCR (Figure 3A) and semi-quantitative RT-PCR (Figure 4C). To determine whether Isl1 expression occurred only with culture *ex vivo*, we checked Isl1 expression in adult whole heart by real-time RT-PCR, and found that Isl1 was indeed expressed in whole heart. This finding is consistent with a recent report by Khattar et al. [16] that showed cells expressing Isl1 protein in adult non-operated murine hearts by immunohistochemical staining. Notably however, the expression level of Isl1 mRNA in CSs was 17-fold higher than in the adult heart (Figure 3A).

To determine the source of Isl1 enrichment in CSs, we analyzed RNA from Sca-1^+^CD45^−^ and CD45^+^ cells sorted from CSs, and found that Isl1 expression in Sca-1^+^CD45^−^ cells was 3-fold higher than in whole CS cells, and that the CD45^+^ fraction did not express Isl1 (Figure 3B). These results suggested that Sca-1^+^CD45^−^ cells derived from CSs are enriched for Isl1^+^ cells, and, as expected, no Isl1^+^ cells were detected among hematopoietic cells of bone marrow origin. In addition, with the exception of CD45^+^ cells, which likely represent inflammatory cells that migrate into the heart post-MI, the proportions of other cell populations in CSs from infarcted heart were not altered compared to non-operated hearts (Figure 4A, B, C).

We investigated whether Isl1 protein expression was detected in the post-MI hearts of “middle aged” mice using immunohistochemical staining and found Isl1^+^ cells in the epicardium at the border of the infarct region at 1 week post-MI (Figure S4). Consistent with our findings, Smart et al [17] also have found significant number of Isl1^+^ cells in the epicardium and subepicardial regions at the border of the infarct scar at 7 days post-MI by genetic tracing and immunohistochemical staining. Together, these indicate that Isl1-expressing cells are present in the post-MI heart of middle aged mice.

### CS cells derived from infarcted myocardium engraft in ischemic myocardium *in vivo*

**Figure 5:**
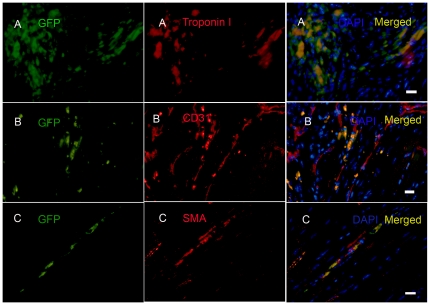
CS cells engraft in ischemic myocardium. CS cells from 1-week post-MI GFP transgenic mice were injected into the peri-infarct zone (PZ) of syngeneic wild type mice 3 days post-MI. Injected cells were detected in the PZ at 25 days after delivery. The surviving cells expressed cardiac Troponin I (A), CD31 (B), and SMA (C). Nuclei were stained with DAPI. Typical results are shown (N = 7). Scale bars = 15 µm in A, B and C.

To determine whether CSs from infarcted myocardium differentiate into cardiac cells *in vivo*, we harvested CSs derived from 1-week post-MI hearts of GFP transgenic mice, injected 10^5^ GFP^+^ cells into the peri-infarct zone (PZ) of syngeneic wild type mice at 3 days post-MI, and analyzed hearts by immunohistochemistry at 25 days post-injection. Numerous GFP^+^ cells were found in infarct and peri-infarct zones (Figure 5A, B and C). Approximately 10% of engrafted GFP^+^ cells expressed cardiac Troponin I, and ∼10% of engrafted GFP^+^ cells expressed either the endothelial cell marker, CD31, or the smooth muscle cell marker, α smooth muscle actin (α-SMA) (Figure 5A, B and C). However, Troponin I^+^GFP^+^ cells lacked the sarcomeric structure seen in typical mature cardiomyocytes, and CD31^+^ or SMA^+^ cells were not incorporated into vascular structures. Nevertheless, our findings demonstrated that the injected CSs cells derived from infarcted heart survived in the ischemic, inflammatory microenvironment for at least 25 days *in vivo*, and expressed markers of nascent cardiac muscle, endothelium, and vascular smooth muscle.

### CSs from infarcted myocardium promote angiogenesis *in vivo*

**Figure 6:**
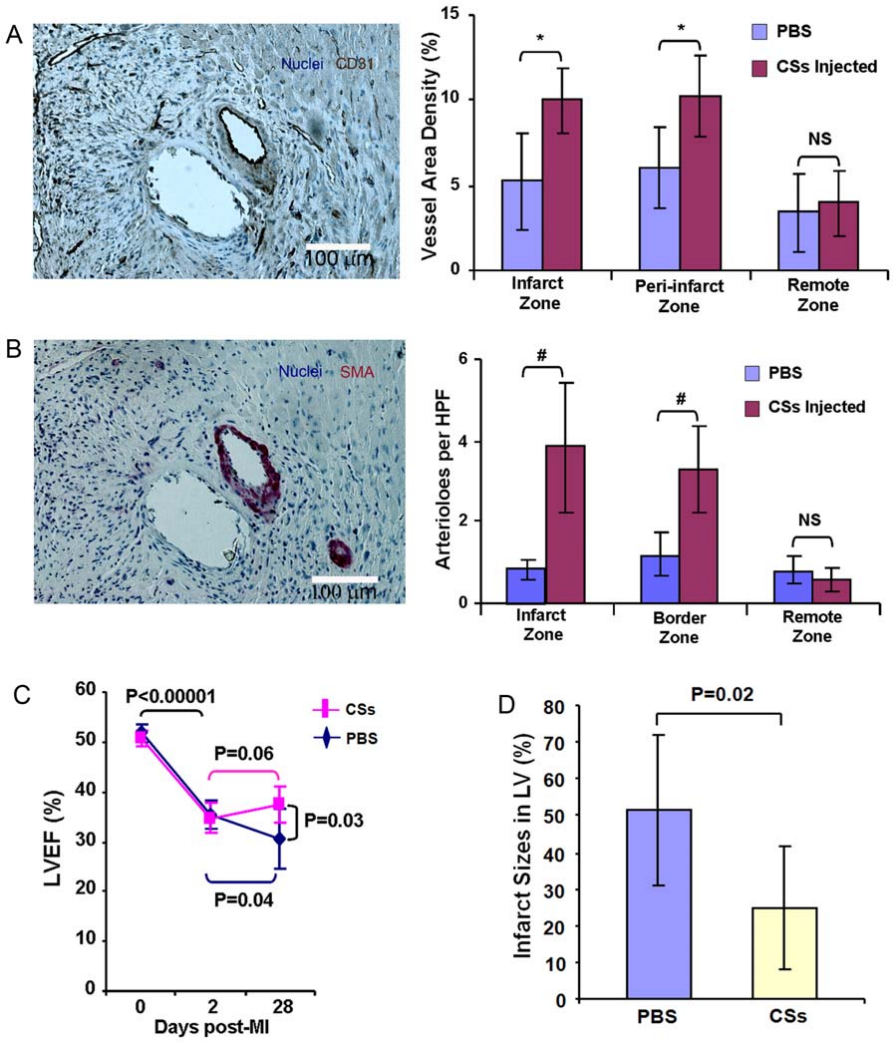
Injected CS cells promote angiogenesis, limited infarct size and improve cardiac function. At 25 days post-injection, engrafted cells resulted in both increased vessel density (A) and numbers of arterioles (B) in the infarct zone and peri-infarct zone, but not remote zone (N = 7). *P = 0.002, #P = 0.0004 (C) LVEF improved with CS cell injection compared to control. Each line represents the mean of one group (N = 6). (D) Mice treated with CS cells had smaller infarct sizes (circumferential extent of scar) compared to control (N = 7).

To determine whether transplantation of CSs promoted angiogenesis in ischemic myocardium, we quantified the capillary and arteriole density in hearts at day 25 post-injection The results showed a greater density of CD31^+^ vessels in the infarct zone (IZ) (10.0±1.9% vs. 5.3±2.8%, P = 0.002) and PZ (10.3±2.4% vs. 6.0±2.4%, P = 0.0004) in CS-injected versus control hearts (Figure 6A). Moreover, the CS-injected group had a significantly higher number of arterioles (SMA^+^) in the IZ and PZ vs. control (3.8±1.6 and 3.2±1.1/high power field (HPF) vs. 0.8±0.2 and 1.2±0.5/HPF, P<0.0005) respectively (Figure 6B). Vessel counts did not differ between the CS-injected and control groups in the remote zone (RZ) (CD31^+^ vessels: 4.0±1.9% vs. 3.4±2.3%, P>0.05; SMA^+^ arterioles 0.6±0.3 vs. 0.8±0.3/HPF, P>0.05) (Figure 6A, B). Because engrafted CD31^+^ and SMA^+^ cells did not contribute to vessel-like structures at this time point, these results suggest that injection of CSs promoted endogenous angiogenesis.

### Injected CSs from infarcted myocardium reduced infarct size and improved cardiac function

To determine whether injection of CSs from infarcted myocardium improved cardiac function in a MI mouse model, we evaluated left ventricular ejection fraction (LVEF) by echocardiography and measured infarct size by histochemical staining at 25 days post-injection. LVEF was significantly reduced from an average of 51.2±1.7% before MI to 35.1±2.9% at 2 days post-MI in both groups, with no significant difference between two groups (P>0.05). At 28 days post-MI (25 days post-injection), LVEF was significantly higher in the CS-injected group (37.5±3.5%) compared to control (30.9±6.6%, P = 0.03) (Figure 6C). Furthermore, we found that the CS-injected group had significantly smaller relative infarct size compared to control (24.8±16.5% vs. 48.4±19.8%, P = 0.02) (Figure 6D and E). These findings suggest that CSs from injured myocardium have a beneficial effect in the MI mouse model.

### Sca-1^+^CD45^-^ cells in CSs have the characteristics of cardiac progenitor cells *in vitro*

In our studies, the Sca-1^+^CD45^-^ subpopulation comprised the largest fraction of CS cells, and contained more Isl1^+^ cells.

To further investigate the origin of Sca-1^+^CD45^-^ in CSs, chimeric mice were generated by transplantation of bone marrow cells isolated from GFP transgenic mice to lethally irradiated C57BL/6 mice. Flow cytometric analysis demonstrated that 88±0.5% of peripheral blood mononuclear cells were GFP^+^ in the chimeric mice 5 months post-transplantation (Figure S5A), indicating stable bone marrow engraftment. About 18.4±4.5% of cells in CSs derived from the no-surgery or 2-week post-MI hearts of chimeric mice co-expressed GFP and CD45 (Figure S5B), indicating they originated from bone marrow. All of the Sca-1^+^CD45^−^ cells were GFP negative (Figure S5C), suggesting that they did not originate from the bone marrow.

**Figure 7:**
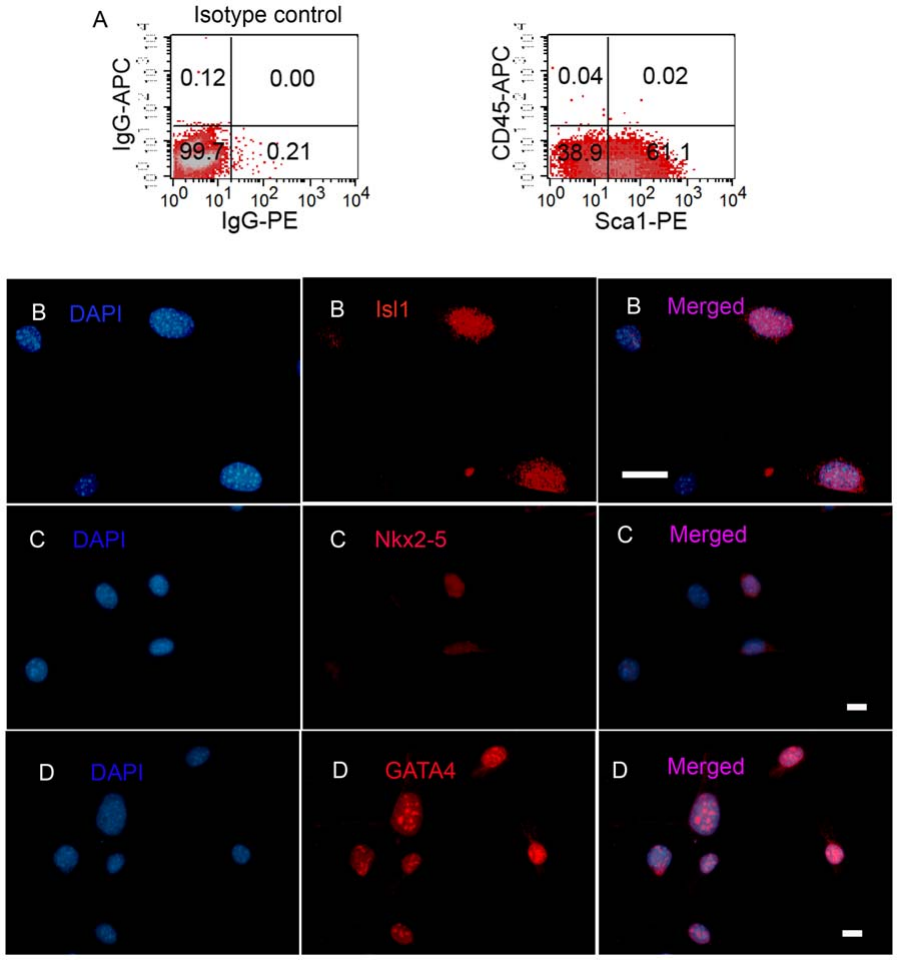
Analysis of cloned Sca-1^+^CD45^-^ cells. (A) Flow cytometry showed that ∼60% of cloned cells were Sca-1^+^CD45^−^. Typical results are shown (N = 6). (B–D) Immunocytochemical staining showed that cloned Sca-1^+^CD45^−^ cells expressed Isl-1 (B), Nkx2-5 (C) and GATA4 (D). Nuclei were stained with DAPI. Scale bar = 35 µm in B3; Scale bar = 15 µm in C3 and D3. Typical results are shown (N = 3).

To further investigate whether these cells play a key role in restoring cardiac function and reducing infarct size, we sorted Sca-1^+^CD45^−^ cells from CSs from 1-week post-MI hearts of adult GFP transgenic mice, and clonally expanded these in culture from single cells. After culturing for 14 days, 5.6% (16/288) of the single cells grew to colonies. About 30% (3/10) of clones grew to >10^6^ cells after 30 days in culture. To determine the cellular composition of cloned cells derived from a single Sca-1^+^CD45^−^ cell, we analyzed the cell-types of cloned cells by FACS after 30 days at passage 4, 15 and 22. FACS analysis showed that 61.8±12.4% of the clonally derived cells was still Sca-1^+^CD45^−^ (Figure 7A), 0.164±0.007% c-kit^+^, 1.7±0.1% CD90^+^, 0.03±0.02% CD133^+^, 1.1±0.6% CD34^+^, 0.6±0.3% CD31^+^ and 0.3±0.2% Flk1^+^. In addition, GATA4, Nkx2-5, and Isl1 were expressed in 60%, 20% and 10% of cloned cells, respectively (Figure 7B, C and D).

**Figure 8:**
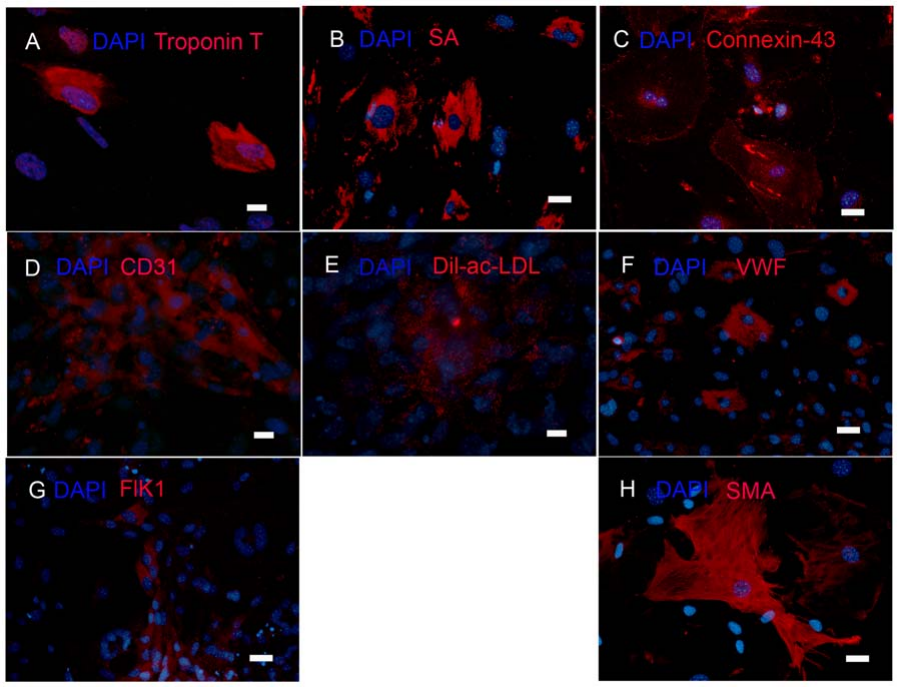
Cloned Sca-1^+^CD45^−^ cells differentiate into cardiac cells *in vitro*. (A–C) After treatment with 5-azacytidine, TGF-β and vitamin C, cloned Sca-1^+^CD45^−^ cells differentiated *in vitro* into cardiomyocytes expressing cardiac Troponin T (A), sarcomeric α-actinin^+^ (SA; B), and connexin-43 (C). (D–H) After treatment VEGF, the cloned cells differentiated into endothelial cells expressing CD31 (D), Von Willebrand Factor (VWF; F), and Flk1 (G), and smooth muscle cells expressing SMA (H). The endothelial cells also demonstrated acetylated LDL uptake (Dil-ac-LDL; E). Nuclei were stained with DAPI. Scale bar = 15 µm in A, D, E, Scale bar = 35 µm in B, C, F, G and H. Typical results are shown (N = 3).

After treatment with 5-azacytidine, transforming growth factor β1 (TGF-β1), and vitamin C [18], cloned Sca-1^+^CD45^−^ cells at 4 and 20 passages differentiated into cardiomyocytes (∼25% of cells expressed sarcomeric α actinin (SA) (Figure 8B), and ∼60% of cells expressed connexin-43 (Figure 8C), the first connexin to be expressed in developing cardiomyocytes [19]). Alternatively, treatment with by vascular endothelial growth factor (VEGF) resulted in differentiation into endothelial cells (∼20% of cells expressed CD31, Von Willebrand Factor (VWF), and FLK-1, and accumulated acetylated low density lipoprotein labeled with 1,1′-dioctadecyl-3,3,3′,3′-tetramethylindo-carbocyanine perchlorate (Dil-ac-LDL)) and smooth muscle cells (∼34% of cells expressed SMA) (Figure 8D, E, F, G and H). Thus, the Sca-1^+^CD45^−^ cells within CSs were clonogenic, multipotent (i.e., formed cardiomyocytes, endothelial cells, and smooth muscle cells), and were capable of long-term self-renewal *in vitro*, all characteristics of cardiac progenitor cells.

To determine whether Isl1 expression persisted in clonally expanded Sca-1^+^CD45^−^ cells, we compared Isl1 transcript levels between primary Sca-1^+^CD45^−^ isolates, clonally expanded Sca-1^+^CD45^−^ cells, and cardiomyocytes differentiated from Sca-1^+^CD45^−^ clones by real-time RT-PCR. We found that expression of Isl1 was not significantly different between primary Sca-1^+^CD45^−^ cells and cloned cells. However, Isl1 expression decreased in cardiomyocytes, consistent with differentiation beyond the progenitor stage (Figure 3C).

### Transplanted Sca-1^+^CD45^−^ cells differentiate into endothelial and smooth muscle cells *in vivo*

**Figure 9:**
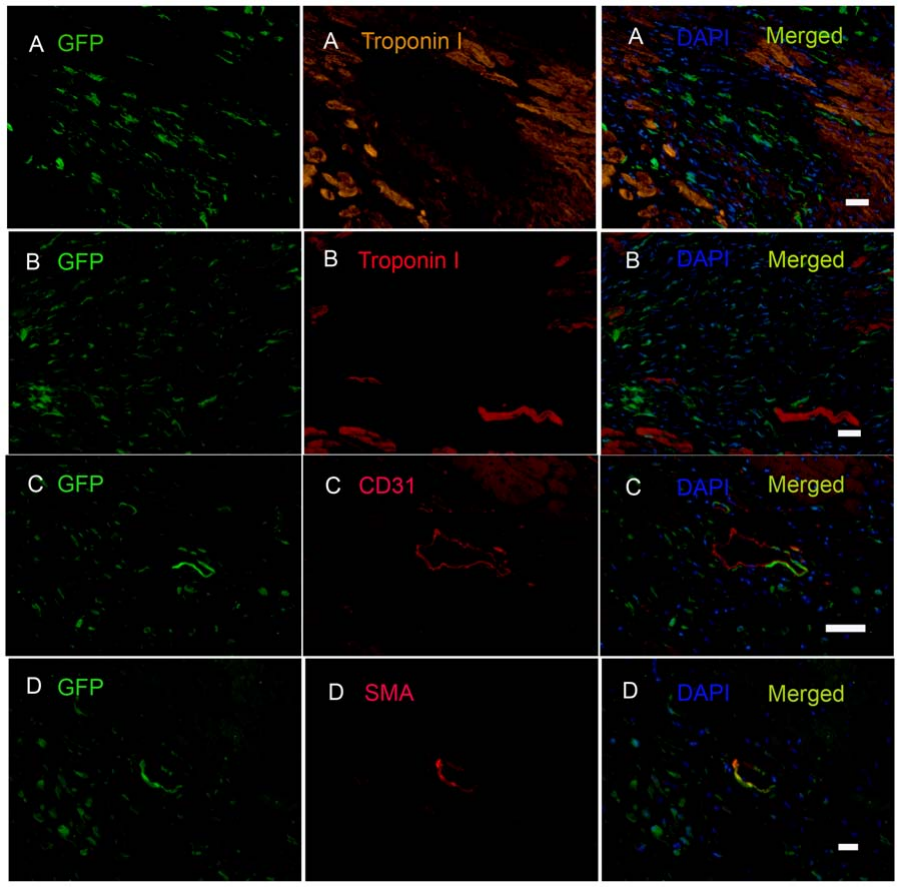
Cloned Sca-1^+^CD45^−^ cells differentiate into endothelial and smooth muscle cells *in vivo*. Cloned Sca-1^+^CD45^−^GFP^+^ cells were injected into the peri-infarct zone (PZ) of infarcted myocardium of syngeneic wild type mice 3 days post-MI. Injected cells were detected by GFP expression 25 days after transplantation, but there was no evidence of cardiac differentiation at this time point (A) (N = 7). At 75 days post-injection (C–D), transplanted cells were detected in the PZ, and expressed CD31 (C) and SMA (D), but not Troponin I (B). Nuclei were stained with DAPI. Scale bar = 35 µm in A, B, C and D. Typical results are shown (N = 2).

To determine whether cloned Sca-1^+^CD45^−^ cells can differentiate after implantation *in vivo*, we injected 10^6^ dissociated cloned GFP^+^ Sca-1^+^CD45^−^ cells into the PZ of syngeneic wild-type mice 3 days post-MI. Twenty five days after injection, the hearts showed numerous implanted cells present in the PZ, but none of the injected cells had differentiated into cardiomyocytes (Figure 9A), endothelial cells and smooth muscle cells at this early time-point after implantation. In contrast, hearts harvested 75 days after cell injection showed that ∼10% of retained transplanted GFP^+^ cells differentiated into CD31^+^ endothelial cells (Figure 9C) or SMA^+^ smooth muscle cells (Figure 9D), but not troponin I^+^ cardiomyocytes (Figure 9B). These findings demonstrate that the cloned Sca-1^+^CD45^−^ cells not only survived in the ischemic microenvironment at 75 days post-injection, but also differentiated into two vascular lineages *in vivo*.

To quantify the level of engraftment and persistence of injected cells in infarcted hearts, cloned Sca-1^+^CD45^−^ (GFP^+^) cells (10^6^) were injected into infarcted hearts of wild type mice. The injected hearts were harvested at 1 hour and 1, 3, 7, 14 and 25 days post-injection. RNA was isolated from whole heart and mRNA expression of GFP was quantified by real-time RT-PCR as a surrogate for the number of engrafted cells. The expression level of GFP in the heart collected 1 hour post-injection was used to represent 100% of injected cells. Approximately 15% and 4% of injected cells were detected in injected heart 3 and 7 days post-injection respectively. Approximately 3% of injected cells were detected 14 to 25 days post-injection (Figure S6).

### Cloned Sca-1^+^CD45^−^ cells promote angiogenesis in ischemic myocardium

**Figure 10:**
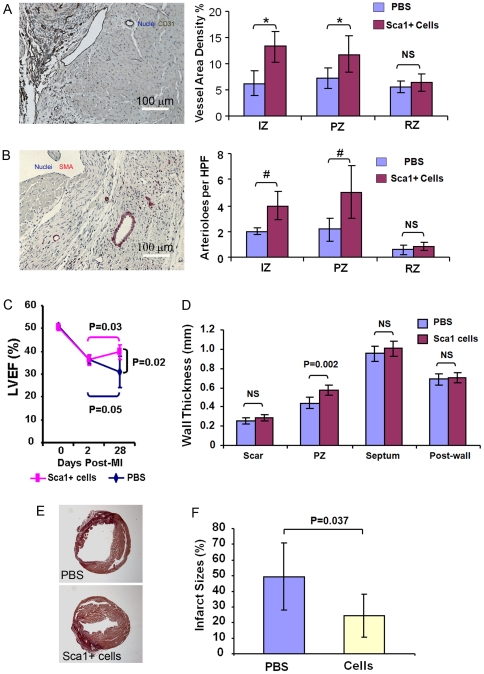
Injected Sca-1^+^CD45^−^ cells promote angiogenesis, limit infarct size and improve cardiac function. At 25 days post-injection of cloned Sca-1^+^CD45^−^ cells, engrafted cells resulted in both increased vessel density (A) and number of arterioles (B) in the infarct zone (IZ) and peri-infarct zone (PZ) but not the remote zone (RZ). *P<0.02, #P<0.009. HPF, high power field at 40X (N = 6). (C) Echocardiography showed that LVEF improved in mice treated with cloned cells compared to control (PBS). Each line represents the mean of one group (N = 6). (D) Left ventricular wall thickness evaluated by the echocardiography showed a significant increase in PZ wall thickness in mice treated with cloned cells versus control (N = 6). (E) Infarct size was determined morphometrically with picosirius red staining (N = 6). (F) Mice treated with cloned cells exhibited smaller infarct sizes than control, as measured by circumferential extent of the scar (N = 6).

To determine whether transplantation of cloned Sca-1^+^CD45^−^ cells promote angiogenesis in ischemic myocardium, we quantified the capillary and arteriole density in hearts 25 days after cell-injection. The results showed that there were more CD31^+^ vessels at the IZ (13.2±2.9% vs. 6.2±2.4%, P = 0.001) and PZ (11.7±3.6% vs. 7.2± 1.9%, P = 0.02) in the cell-injected group vs. control (Figure 10A). Moreover, the cell-injected group had a significantly higher number of SMA^+^ arterioles in the IZ (3.9±1.1 vs. 1.9±0.3/HPF, P = 0.001) and PZ (5.0±2.0 vs. 2.1±0.9/HPF, P = 0.009) vs. control (Figure 10B). Cell-injection had no effect on vascular density in the RZ (CD31^+^ vessels: 6.4±1.7% vs. 5.7±1.1%, P>0.05; SMA^+^ arterioles 0.8±0.3 vs. 0.7±0.3/HPF, P>0.05) (Figure 10A, B). However, none of the vessels contained GFP^+^ cells 25 days after cell-injection, suggesting that cloned Sca-1^+^CD45^−^ cells promoted endogenous angiogenesis by paracrine mechanisms, rather than by direct participation in new blood vessel formation at this time point. Together, it is likely that transplanted cloned Sca-1^+^CD45^−^ cells induce angiogenesis through both paracrine effects and transdifferentiation. However, the small number of vessels with GFP^+^ cells detected 75 days post-transplantation suggests that angiogenesis is induced mainly through paracrine effects of the engrafted cells.

### Cloned Sca-1^+^CD45^−^ cells reduce apoptosis of cardiomyocytes

To evaluate the effects of Sca-1^+^CD45^−^ cells injection on cardiomyocyte apoptosis, we used terminal deoxynucleotidyl transferase dUTP nick end labeling (TUNEL) and co-staining troponin I in the hearts 25 days after cell-injection. Cell injection resulted in significant reduction in the number of TUNEL^+^/troponin I^+^ cells in PZ compared to control (0.25±0.17 vs. 1.08±0.35/low power field (LPF), P<0.05) (Figure S7). The number of TUNEL^+^/troponin I^+^ cells did not differ between cell-injected and control groups in IZ and RZ (Figure S7).

### Cloned Sca-1^+^CD45^−^ cells reduce infarct size and improve cardiac function

To determine whether the cloned Sca-1^+^CD45^−^ cells alone improve cardiac function in the MI mouse model, we evaluated LVEF by echocardiography and measured infarct size by histochemistry 25 days after cell injection. LVEF was significantly reduced from an average of 51.2±1.5% before MI to 36.3±2.0% at 2 days post-MI in both groups, with no significant difference between the two groups (P = 0.9). At 28 days post-MI (25 days post-injection), LVEF was significantly higher in the cell-injected group compared to control (39.7±3.2% versus 30.9±6.6%, P = 0.02) (Figure 10C). In addition, the myocardium was significantly thicker in the peri-infarct wall in the cell injected group compared to control (0.57±0.05 vs. 0.44±0.06 mm, P = 0.002), but there was no statistically significant difference in the thickness of the posterior wall (0.70±0.05 vs. 0.69±0.06 mm, P = N.S.), septum (1.0±0.08 vs. 0.95±0.08 mm, P = N.S.) or scar (0.29±0.03 vs. 0.25±0.03 mm, P = N.S.) between the groups (Figure 10D). The cell-injected group had significantly smaller infarcts compared to control (24.4±13.8% vs. 49.4±21.5%, P = 0.037) (Figure 10E, D). These studies suggest that the cloned Sca-1^+^CD45^−^ cells confer the therapeutic benefits of CSs in the mouse MI model.

## Discussion

In this study, we have shown that: 1) there is a significant increase in the proliferative capacity of CS-forming cells isolated from the “middle aged” heart following acute MI resulting in a significant rise in the number of CSs *in vitro*; 2) this increase is time-dependent and is most pronounced within the first week post-MI in the animal model studied; 3) transplanted CSs from infarcted myocardium engraft in ischemic myocardium, improve cardiac function, and promote endogenous angiogenesis; 4) adult heart contains Isl1^+^ cells and Isl1 expression in CSs is 17-fold higher than in total adult cardiac tissue; 5) Isl1 expression in the Sca-1^+^CD45^−^ subpopulation within CSs is 3-fold higher than in total CSs; 6) Sca-1^+^CD45^−^ cells in CSs can be cloned, expanded, and have the characteristics of multipotent cardiac progenitor cells *in vitro*; and 7) after injection into ischemic myocardium, cloned Sca-1^+^CD45^−^ cells not only survive long-term, but also differentiate into endothelial and smooth muscle cells, promote endogenous angiogenesis, reduce cardiomyocyte apoptosis, reduce infarct size, and improve cardiac function. Of special note, these experiments have been performed in middle-aged mice, rather than in young adults, to simulate a more clinically relevant disease model.

Previous studies [20] have shown that the number of Sca-1^+^ cardiac progenitor cells increases post-MI. Our data agrees with these reports and further shows that the potential for CSs generation *in vitro* is highly time dependent post-MI. The number of CSs from 1- and 2-week post-MI hearts greatly increases compared to uninjured hearts, and this increase is attenuated by 4 weeks post-MI. This suggests that acute MI induces the proliferation of cardiac progenitor cells, and this increase in proliferation gradually dissipates over a 4-week period post-MI. Therefore early acquisition of tissue from post-MI hearts would facilitate higher yields of CSs *in vitro*. However, the optimal timing in humans may be different than the ones reported in our study and future research is necessary to better define the ideal timing of tissue acquisition in patients post-MI.

A previous report [21] showed that stem cell niches distribute preferentially to the apex and atria of the heart, where the wall stress is relatively low. MI may affect their distribution. Our results show that various regions of 1-week post-MI hearts have similar abilities to produce CSs. This suggests that although MI mainly affects the LV, the CS-forming cells throughout the heart are activated post-MI. As such, taking tissue from any region of the heart appears to yield similar numbers of CSs per unit of tissue. The mechanism by which MI increases CSs production is worthy of further investigation. Importantly, the septum and right ventricle yield the same numbers of CSs. Thus, percutaneous right ventricular endomyocardial biopsy, as is performed routinely for other clinical indications, could potentially be used to generate CSs in the post-MI setting.

It has been previously reported that CSs from non-infarcted hearts could differentiate into cardiac cells and preserve cardiac function[7]. Whether CSs derived from early stage post-MI hearts have the same abilities has not been demonstrated until now. Our results show for the first time that the CS cells obtained from 1-week post-MI hearts engraft in ischemic myocardium and restore cardiac function at 25 days post-injection *in vivo*. However, we did not find evidence for differentiation of these cells into mature cardiomyocytes or new vessels. Our data demonstrate that the injected CSs promoted angiogenesis *in vivo*, suggesting that engrafted CSs cells likely have a paracrine, pro-angiogenic effect in the ischemic myocardium. Secreted VEGF from engrafted CSs may contribute to angiogenesis in the infarcted hearts [22]. This paracrine effect may play an important role in attenuating adverse LV remodeling and preserving cardiac function.

Recent reports have found Isl1^+^ cells in adult mouse, rat and human hearts [16], [17], [23], [24]. Our results not only confirm the presence of Isl1^+^ cells in adult murine hearts, but we show that these can be efficiently isolated and expanded by culturing Sca-1^+^CD45^−^ cells from CSs. Since Isl1 is not expressed on the cell surface, it has been difficult to isolate and purify these cells by immune selection. However, we now demonstrate that isolating Sca-1^+^CD45^−^ cells from CSs results in an enriched population of Isl1^+^ cardiac progenitors for autologous cardiac cell-therapy.

While cloned Sca-1^+^CD45^−^ cells improved cardiac function post-MI in transplanted mice, we did not find evidence for differentiation of these cells into cardiomyocytes *in vivo*. One possible explanation is that the cloned Sca-1^+^CD45^−^ cells may need a longer time to differentiate into cardiomyocytes *in situ*. This is supported by the observation that there was no differentiation seen from cloned cells in the first 25 days, while endothelial and smooth muscle cell differentiation occurred only after 75 days. Another possible explanation is that there may be subpopulations of Sca-1^+^CD45^−^ CS cells with distinct differentiation capacities. Whether resident cardiac progenitor cells in adult hearts are capable of cardiomyogenic differentiation *in vivo* remains controversial [14], [25]–[27]. The therapeutic effects of CS-derived Sca-1^+^CD45^−^ cells *in vivo* do suggest, however, that these cells might be responsible for the overall effects of CSs. Since the Sca-1^+^CD45^−^ cells can be clonally expanded *in vitro*, they provide a feasible approach to rapidly generating therapeutic quantities of cardiac progenitor cells.

A recent report has suggested that CSs are composed of fibroblasts, and not cardiac progenitor cells [13]. Although many fibroblasts grow out from the cardiac explant during the first stage of culture, we demonstrate here that our CSs contain cardiac progenitor cells that are capable of clonal expansion and multi-lineage cardiac differentiation. Furthermore, our demonstration that cloned Sca-1^+^CD45^−^ cells have a beneficial therapeutic effect, similar to heterogeneous CSs, argues strongly against the hypothesis that fibroblasts are the major contributors to cardiac repair in CSs.

There are several limitations to this study. First, under the experimental conditions used, we did not find evidence for differentiation of cloned Sca-1^+^CD45^−^ cells into cardiomyocytes *in vivo*. It is possible that a longer follow-up period might be required for this differentiation to be observed *in vivo*. Despite this, we have demonstrated that these cells do indeed have “progenitor” characteristics given their ability to differentiate into other cell types both *in vivo* and *in vitro*. Second, we show increased angiogenesis and reduced cardiomyocyte apoptosis after injection of cloned Sca-1^+^CD45^−^ cells. However, given our experimental conditions, we are unable to address whether the injected cells recruit and influence endogenous cardiac progenitors *in vivo*. Recently, using a genetic lineage mapping approach, Loffredo et al [28] have reported new cardiomyocyte formation in infarcted hearts derived from endogenous cardiac stem cells 8 weeks after injecting with murine bone marrow c-kit^+^ cells. There are several possible explanations for the consistent improvements in ventricular function including: reduction in cardiomyocyte apoptosis [22], [29], [30], prevention of infarct scar expansion by mechanically stiffening the infarct zone, facilitation of hypertrophy of the border zone cardiomyocytes by enhanced angiogenesis [22], [29]–[31]. Other groups have reported stimulation of the endogenous adult cardiomyocytes to re-enter cell cycle and divide [32] and recruitment and/or activation of resident cardiac progenitors [22], [28], [32] as possible additional mechanisms. Each of these mechanisms has been implicated in improved cardiac function seen with cell therapy. However, the exact contribution of each of these mechanisms to the overall benefit of therapy remains the focus of intense research.

In summary, our data suggest that the cloned Sca-1^+^CD45^−^ cells derived from CSs from post-MI hearts are enriched in Isl1^+^ progenitors, have the characteristics of progenitor cells, and are an attractive source of autologous cells for myocardial therapy post-MI.

## Materials and Methods

### Animals

C57BL/6J mice and C57BL/6J GFP transgenic mice with chicken α-actin promoter driving EGFP expression were purchased from the Jackson Laboratory (Bar Harbor, Maine). All animals were housed in the animal care facility at The University of California, San Francisco (UCSF), and all experiments were approved by, and conducted in accordance with the guidelines of, the Institutional Animal Care and Use Committee of UCSF (Approval number: AN078431).

### Myocardial infarction model

Nine month-old, male C57BL/6J mice were used for all experiments to simulate “middle-aged” subjects. Mice underwent total permanent ligation of the left anterior descending coronary artery (LAD) to induce MI, and hearts were collected 1, 2, and 4 weeks post-MI (n = 6/group). Hearts were also harvested from animals that had undergone sham operation or no surgery (n = 6/group). The surgical procedure for MI has been previously described [29], [33], [34]. Briefly, mice were anesthetized using isoflurane, intubated and ventilated. A midline thoracotomy incision was made to expose the heart for surgery. LAD was ligated permanently. Sham operation was performed by passing a suture under the LAD and removing it without ligation.

### Generating chimeric mouse

Bone marrow cells were harvested from 8–10 week old GFP transgenic mice and transplanted into lethally irradiated (9.5 Gy) 2 month -old C57BL/6 mice through tail vein injection (2x10^6^ nucleated unfractionated cells per mouse). The expression of GFP by peripheral blood mononuclear cells was analyzed by FACS 5–6 months later. CSs were isolated from no-surgery or 2-week post-MI hearts of chimeric mice.

### Cell transplant studies

Nine month-old, male C57BL/6J GFP transgenic mice (n = 12) were used as CSs donors. One week post-MI, hearts were harvested and used to generate CSs for injection. CSs were dissociated into single cell suspension by Blendzyme 4 and resuspended with phosphate buffered saline (PBS). Alternatively, cloned Sca-1^+^CD45^−^ cells were dissociated into single cell suspension by trypsin and also resuspended with PBS. These cells in 10 µl PBS were injected into the hearts of 3-day post-MI mice by ultrasound-guided injection, a technique developed and reported by our laboratory [29], [34]. PBS only was injected into infarcted hearts as control. Two month-old, wild-type C57BL/6J mice were used as recipients. The recipient mice received either CS cells (n = 7), Cloned Sca-1^+^CD45^−^ cells (n = 8) or PBS (n = 12), and hearts were harvested 25 days post-injection or 75 days post-injection (Sca-1^+^CD45^−^ cell injections only). For studying the retention of injected cloned Sca-1^+^CD45^−^ cells, eighteen wild-type mice were used as recipients and whole hearts were harvested 1 hour and 1, 3, 7, 14 and 25 days post-injection (n = 3/each time point) for RNA isolation.

### Cardiosphere culture

CSs were generated using the method described by Messina et al. [7] with minor modification. The whole heart was removed from the mice and cut into 1–2 mm^3^ pieces. After being washed with Ca^++^ Mg^++^ free PBS and digested three times, 5 minutes each at 37^0^C with 0.25% trypsin (Invitrogen, Carlsbad, CA) and 0.1% collagenase D (Roche Diagnostics, Indianapolis, IN), the tissue pieces were cultured as “explants” on fibronectin (Sigma, St Louis, MO) coated 6-well plates, 2 wells for each heart in IMDM medium with 10% FBS and 0.1 mM β-mercaptoethanol at 37^0^C with 5% CO_2_. A layer of fibroblast-like cells grew from explants, over which small, round phase-bright cells (CS-forming cells) appeared 2 to 4 weeks after initiating the culture. Once the fibroblast-like cells grew to 90% confluence determined visually, the cells surrounding the explants were harvested by two washes with PBS, one wash with 0.53 mmol/L EDTA and one wash with 0.05% trypsin (Invitrogen) at room temperature. The harvested cells were filtered by 70 mm cell strainer (BD Biosciences, San Jose, CA), and then cultured at a density of 1x10^5^ cells/ml in each well of 24-well plates coated with Poly-D-Lysine (BD Biosciences) in cardiosphere growth medium (CGM), which included 35% IMDM, 65% DMEM-F12, 3.5% FBS, 0.1 mM β-mercaptoethanol, 2% B27 (Invitrogen), 10 ng/ml EGF (R&D systems), 20 ng/ml bFGF (R&D systems), 40 nmol/L thrombin (R&D systems) and 4 nmol/L cardiotrophin (R&D systems). The number of CSs in each well was counted on the 7^th^ days after the CS-forming cells were plated.

### Flow cytometry and cells sorting

CSs were dissociated into single cell suspension by Blendzyme 4 (5.6u/ml) (Roche). The following phycoerythrin (PE) or allophycocyanin (APC) conjugated rat anti-mouse antibodies and conjugated isotype-matched control antibodies were used: Sca-1-PE, c-kit-PE, CD133-PE, CD34-PE, CD45-APC, Flk-1-APC and CD31-APC (eBioscience). The cells were incubated with antibodies for 25 min on ice, washed with PBS containing 0.2% BSA, and analyzed by FACSCabilur with CellQuest software (BD Biosciences).

For cell sorting, the dissociated CS cells from hearts of 1-week post-MI GFP transgenic mice were stained by the following antibodies: Sca-1-PE and CD45-APC. The Sca-1^+^CD45^−^ cells were sorted by FACSAria with FACSdiva software (BD Biosciences) and were dropped into a 96-well plate, one cell/well, on top of mitomycin-C treated murine embryonic fibroblast cells (Millipore, New Jersey). The cells were then cultured with CGM at 37^0^C with 5% CO_2_. For isolating RNA, sorted Sca-1^+^CD45^−^ cells or CD45^+^ cells from CSs were collected into a tube with 1 ml of CGM respectively.

### Directed differentiation *in vitro*

Cloned Sca-1^+^CD45^−^ cells were loaded into chamberslides coated with gelatin at 15,000 cells/cm^2^ in differentiation medium, treated by 5-Azacytidine (5 mM) for 3 days, and then we added TGF-β1 (1 ng/ml) and vitamin C (0.1mM) for three weeks to induce cardiomyocyte differentiation[18]. The cells were treated with VEGF (20 ng/ml) in IMDM medium with 10% FBS for 2 weeks for endothelial and smooth muscle cells differentiation [18].

### Immunocytochemistry

The cells cultured in chamberslides were washed with PBS, fixed with cold methanol for 5 min or 4% paraformaldehyde/PBS for 15 min, and blocked with Dako antibody diluent (DakoCytomation, Carpinteria, CA) for 1 hour. When using mouse derived monoclonal antibody, we also used Rodent Block M (Biocare Medical, Concord, CA) blocking for 30 min. The cells were incubated with the following primary antibodies diluted in Dako antibody diluent at 4^0^C overnight: rabbit anti-Nkx2-5, GATA4 (Santa Cruz Biotechnology, Santa Cruz, CA), mouse anti-Isl-1 (39.4D5) (Developmental Studies Hybridoma Bank, Iowa City, IA), mouse anti-α SMA (Sigma), mouse anti-Troponin T (Thermo Fisher Scientific, Fremont, CA), mouse anti-SA (Abcam, Cambridge, MA), rabbit anti-connexin-43 (Sigma), mouse anti-CD31 (Abcam) and rabbit anti-VWF (Abcam). The cells were then incubated with the Alexa Fluor 546 labeled goat anti-rabbit antibody or goat anti-mouse antibody (Invitrogen) at room temperature for 1 hour. The slides were mounted with ProLong Gold antifade reagent with DAPI (Invitrogen) and viewed with a Nikon E800 fluorescence microscope using Openlab software (Improvision, Lexington, MA).

### Acetylated-LDL uptake assay

Acetylated low density lipoprotein labeled with Dil-ac-LDL (Invitrogen) was added into the medium with cells at 2ug/ml as final concentration and incubated at 37^0^C with 5% CO_2_ for 1 hour. The medium was removed. The cells were washed with PBS and fixed with 4% paraformaldehyde/PBS for 15 min. The slides were mounted and viewed same as immunocytochemical staining.

### RT-PCR and Real-time RT-PCR

The total RNA from CSs and tissues were isolated by TRIzol reagent (Invitrogen). cDNA was generated from 0.3 µg of total RNA by using SuperScript III First-Strand Synthesis kit (Invitrogen). RT-PCR was performed using 1 µl of cDNA and Advantage 2 PCR kit (Clontech, Mountain View, CA) with the following program: 95°C 3 min, (95°C 30 s– 68°C– 3 min)×30 cycles, 68°C 10 min. PCR products were separated on 2% agarose gel. Every pair of PCR primers was designed to span one or several introns to distinguish the signals amplified from genomic DNA contamination. The primers sequence of Nkx2-5, GATA4, Flk-1, SMA and internal control hypoxanthine phosphoribosyltransferase (HPRT) are from previous publications [4], [35], [36].

The total RNA from sorted cells was isolated and the cDNA was generated by Taqman Gene Expression Cells-to-Ct kit (Applied Biosystems, Foster City, CA, USA). The primers and probe for murine Isl1 and HPRT were purchased from Applied Biosystems. The real-time PCR were performed by ABI PISM 7300 (Applied Biosystems) using Taqman Master Mix (Applied Biosystems) in duplicates and the average threshold cycles (CT) of duplicate were used to calculate the relative value of Isl-1 in different cells and tissues. The CT for HPRT was used to normalize the samples. Expression of Isl1 mRNA relative to HPRT mRNA was calculated based on the CT, ΔCT_Isl1_ = CT_Isl1_-CT_HPRT_. The relative values of Isl1 were calculated as 2^−ΔCTIsl1^.

For studying the retention of injected cloned GFP^+^ Sca-1^+^CD45^−^ cells, total RNA from whole heart was isolated by TRIzol, genomic DNA was removed from total RNA by RNeasy Mini Kit with RNase-free DNase (Qiagen) and 25-ng cDNA was used for real-time PCR. The sequences of primers and probes for GFP and histone 3.3A were as previously published [29]. Expression of GFP mRNA relative to histone 3.3A mRNA was calculated based on the CT, ΔCT_GFP_ = CT_GFP_-CT_histone_. The relative values of GFP were calculated as 2^−ΔCTGFP^.

### Tissue analysis

Tissue was analyzed by two blinded reviewers. Mice were sacrificed 25 days post-injection of cells (28 days post-MI) or 75 day post-injection. The hearts were arrested in diastole with KCl, perfusion and fixed with 10% formalin, embedded in paraffin, cut into 5 mm sections and blocked with Dako antibody diluent for 1 hour. When using mouse or rat derived monoclonal antibody, we also incubated the sections with Rodent Block M or R blocking for 30 min. To detect GFP and troponin I double positive cells, sections were stained with anti-troponin I (Abcam) and rabbit anti-GFP (Invitrogen) overnight at 4°C. Alexa Fluor 546 goat anti mouse IgG and Alexa Fluor 660 goat anti rabbit were used as secondary antibodies (Invitrogen). Detection of GFP and CD31 double positive cells were stained with rat anti-CD31 (Biocare Medical) and Alexa Fluor goat anti rat 546 were used secondary antibodies. GFP and α-SMA double positive cells were stained with mouse anti α-SMA and without prior antigen retrieval but otherwise followed the steps described above. The slides were mounted with ProLong Gold antifade reagent with DAPI and viewed with a Nikon E800 fluorescence microscope using Openlab software.

In order to assess vascular density in the hearts, the sections from mid-ventricular level were stained by antibodies of rat anti-CD31 and mouse anti- α-SMA at room temperature for 1–2 hours. A CD31 signal was detected using a Rat on Mouse HRP-Polymer kit (Biocare) and 3,3′Diaminobenzidine (DAB) (Biocare). α-SMA signal was detected by a MM AP-Polymer kit (Biocare) and a Vulcan Fast Red Chromogen kit (Biocare) for color development. The slides were mounted and observed as described above. ImagePro Plus 6.0 software (MediaCybernetics, Bethesda, MD) was used to analyze the percentage area occupied by CD31 positive vessels. The number of arterioles, defined as vessels with CD31^+^ endothelial cells surrounded by α-SMA^+^ smooth muscle cells, per HPF in each region was counted [29].

For Isl1 staining, the hearts were perfused and fixed with 4% paraformaldehyde overnight, equilibrated with 20–30% sucrose and frozen in OCT for tissue sectioning using a cryostat. The sections were blocked with Rodent Block M and Dako antibody diluent for 30 min respectively, stained with mouse anti-Isl-1 (39.4D5) overnight at 4°C and Alexa Fluor 546 goat anti mouse IgG used as secondary antibody.

TUNEL staining was performed with ApopTag® Plus Peroxidase *In Situ* Apoptosis Detection Kit (Chemicon, Temecula, CA) according to manufacture's protocol and DAB was used for color development. For co-staining troponin I, the sections from mid-ventricular level were treated with denature solution (Biocare), blocked with Rodent Block M and then incubated with mouse anti-troponin I. The mouse-on-mouse alkaline phosphatase polymer (Biocare) was used as secondary antibody. Vulcan Fast Red Chromogen kit was used for color development. Finally the sections were counterstained with hematoxylin. TUNEL-positive cardiomyocytes were defined by the presence of both DAB nuclear staining and completely surrounded by troponin I staining.

In order to assess the size of infarct scar, the sections from mid-ventricular level (mid-papillary) were stained by picosirius red. The scar was stained as dark red. The slides were mounted and viewed same as above. All histological sections were examined with a Nikon Eclipse E800 microscope using a 1x objective with the use of Openlab software (Improvision, Lexington, MA). To assess the circumferential extent of the infarct, the epicardial and endocardial infarct lengths, epicardial and endocardial circumferences of LV were traced manually using the ImagePro Plus 6.0 software. Epicardial infarct ratio was obtained by dividing the epicardial infarct length by the epicardial circumference of LV. Endocardial infarct ratio was calculated by dividing the endocardial infarct length by the endocardial circumference of LV. The circumferential extent of the infarct scar was calculated as [(epicardial infarct ratio + endocardial infarct ratio)/2]×100.

### Echocardiography

Echocardiography was accomplished under isoflurane anesthesia with the use of a Vevo-660 (VisualSonic, Toronto) equipped with a 30 MHz transducer. Echocardiograms were obtained at baseline, 2 days post-MI (before injection), and day 28 post-MI. We measured LVEF and wall thickness. Wall thickness was measured at the apical anterior wall (infarct wall thickness) and at the mid-anterior segment (peri-infarct wall thickness) separately on the parasternal long-axis view; posterior wall thickness was obtained at the papillary muscle level. Three cycles were measured for each assessment and average values were obtained [29], [33]. Echocardiograms were analyzed by a blinded reviewer.

### Statistical analysis

One way ANOVA with Fisher's post hoc test was used to analyze the difference among multiple groups. Student's t test was used to analyze differences between two groups. Values were expressed as mean±SD unless otherwise specified, with P<0.05 considered significant. SPSS 15.0 software was used to conduct all statistical analysis.

## Supporting Information

Figure S1 **CS culture.** (A) Typical explants of cardiac tissue, one day after placing into culture. (B) CS-forming cells are seen as small, round, phase-bright cells arising from the fibroblast-like monolayer around the attached explant after 14 days. (C) CSs appear 3 days after the CS-forming cells are re-plated in separate wells. Typical results are shown (N = 12). Scale bar = 200 µm.
(TIF)

Figure S2 **Cellular composition of CS-forming cells from mouse hearts.** (A) Flow cytometric analysis of Sca-1, CD45 and c-kit expression in disaggregated CS cells (N = 5). (B) Bar graph showing the profile of progenitor cell markers in CSs by FACS (N = 5). Data are shown as mean±SEM.
(TIF)

Figure S3 **CSs generated from different cardiac regions.** Different cardiac regions generated similar number of CSs per milligram of tissue at 1 week post-MI. LV, left ventricle excluding scar; RV, right ventricle; LA, left atrium and RA, right atrium. Data are shown as mean±SEM (N = 4).
(TIF)

Figure S4 **Isl1^+^ cells in post-MI heart.** After 7 days post-MI, Isl1^+^ cells were detected in epicardium at the border of infarct region by immunohistochemical staining in 9 month old mice at lower power (scale bar = 35 µm) (A) and higher power (scale bar = 100 µm) (B) (N = 3).
(TIF)

Figure S5 **Sca-1^+^CD45^-^ cells in CSs from chimeric mouse are GFP negative.** Flow cytometric analysis GFP^+^ cells in peripheral blood mononuclear cells in wild type mouse (A1) and in chimeric mouse 5 months post-transplantation (A2). FACS showed CD45^+^ cells in CSs from wild type mouse (B1) and CD45^+^GFP^+^ cells in CSs from chimeric mouse (B2). FACS showed Sca-1^+^CD45^-^ cells in CSs from chimeric mouse were GFP negative, whereas Sca-1^+^CD45^+^ cells were GFP positive (C). Typical results are shown (N = 4).
(TIF)

Figure S6 **The level of engraftment and persistence of injected cells in infarcted hearts.** Real-time RT-PCR (Taqman) was used to compare GFP expression in hearts injected with cloned Sca-1^+^CD45^-^GFP^+^ cells. The injected hearts were harvested at 1 hour and 1, 3, 7, 14 and 25 days post-injection. Results show as GFP mRNA expression relative to histone 3.3A. The expression level of GFP in the heart collected 1 hour post-injection was used to represent 100% of injected cells. H: hour; D: day. Typical results are shown (N = 3).
(TIF)

Figure S7 **Injected Sca-1^+^CD45^-^ cells reduce cardiomyocyte apoptosis.** Typical image showed TUNEL^+^/Troponin I^+^ cells (black arrow) (A). Sca-1^+^ cell injection resulted in a significant reduction of TUNEL^+^/Troponin I^+^ cells in the peri-infarct zone (PZ), but not in the infarct zone (IZ) and remote zone (RZ) compared to the control group 25 days post-injection (B). TnI, troponin I; TUNEL, terminal deoxynucleotidyl transferase dUTP nick end labeling; CM, cardiomyocyte; LPF, low power field (20 x magnification); Data are shown as mean±SEM (N = 6). #<0.05.
(TIF)
