# Supplementary figures and images for: Sca-1+ Cardiosphere-Derived Cells Are Enriched for Isl1-Expressing Cardiac Precursors and Improve Cardiac Function after Myocardial Injury
Source: PLoS One. 2012 Jan 17;7(1):e30329. doi: 10.1371/journal.pone.0030329 (PMC3260268; doi:10.1371/journal.pone.0030329)

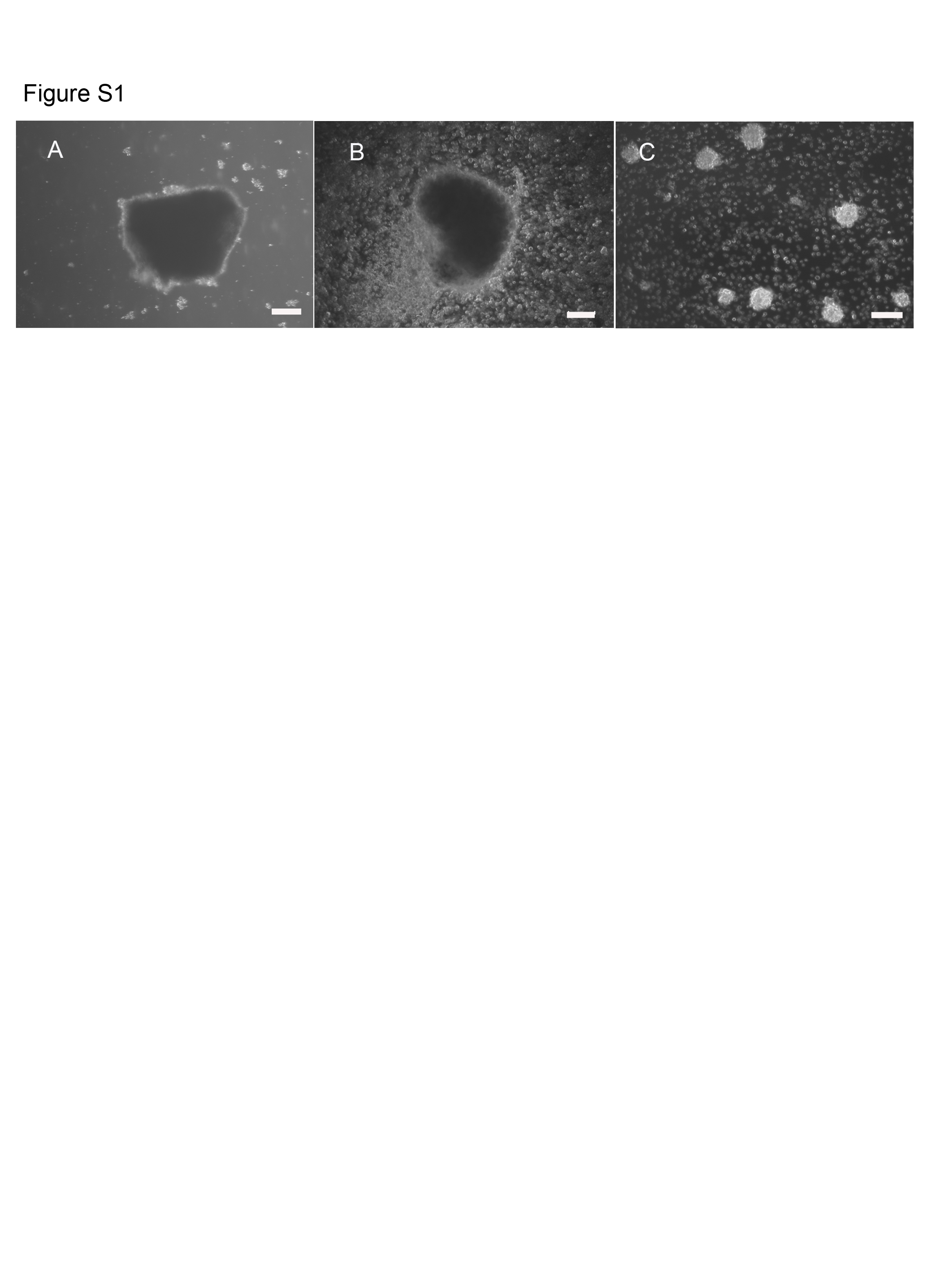

Supplement: Figure S1 — CS culture. (A) Typical explants of cardiac tissue, one day after placing into culture. (B) CS-forming cells are seen as small, round, phase-bright cells arising from the fibroblast-like monolayer around the attached explant after 14 days. (C) CSs appear 3 days after the CS-forming cells are re-plated in separate wells. Typical results are shown (N = 12). Scale bar = 200 µm. (TIF) [file pone.0030329.s001.tif]

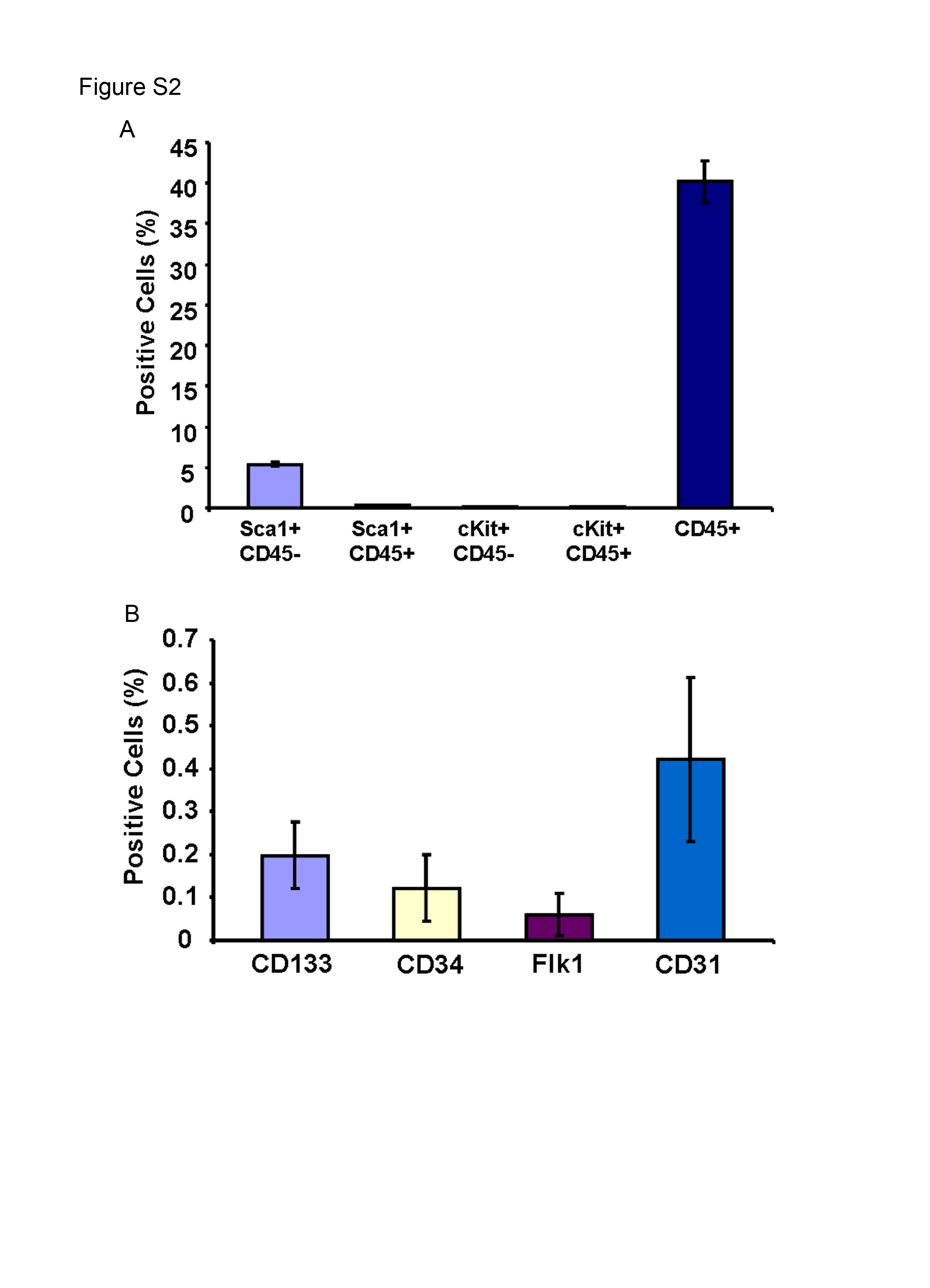

Supplement: Figure S2 — Cellular composition of CS-forming cells from mouse hearts. (A) Flow cytometric analysis of Sca-1, CD45 and c-kit expression in disaggregated CS cells (N = 5). (B) Bar graph showing the profile of progenitor cell markers in CSs by FACS (N = 5). Data are shown as mean±SEM. (TIF) [file pone.0030329.s002.tif]

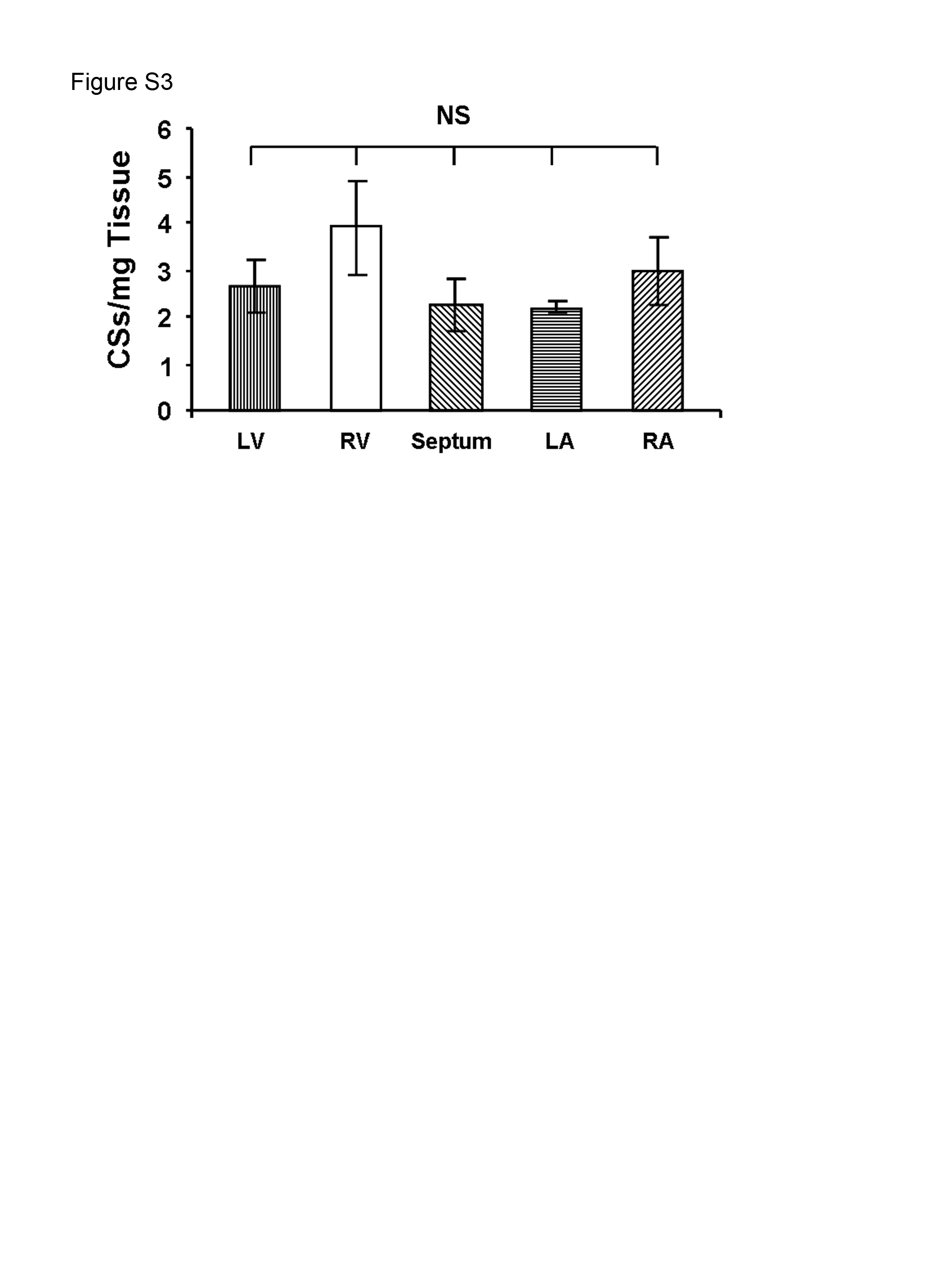

Supplement: Figure S3 — CSs generated from different cardiac regions. Different cardiac regions generated similar number of CSs per milligram of tissue at 1 week post-MI. LV, left ventricle excluding scar; RV, right ventricle; LA, left atrium and RA, right atrium. Data are shown as mean±SEM (N = 4). (TIF) [file pone.0030329.s003.tif]

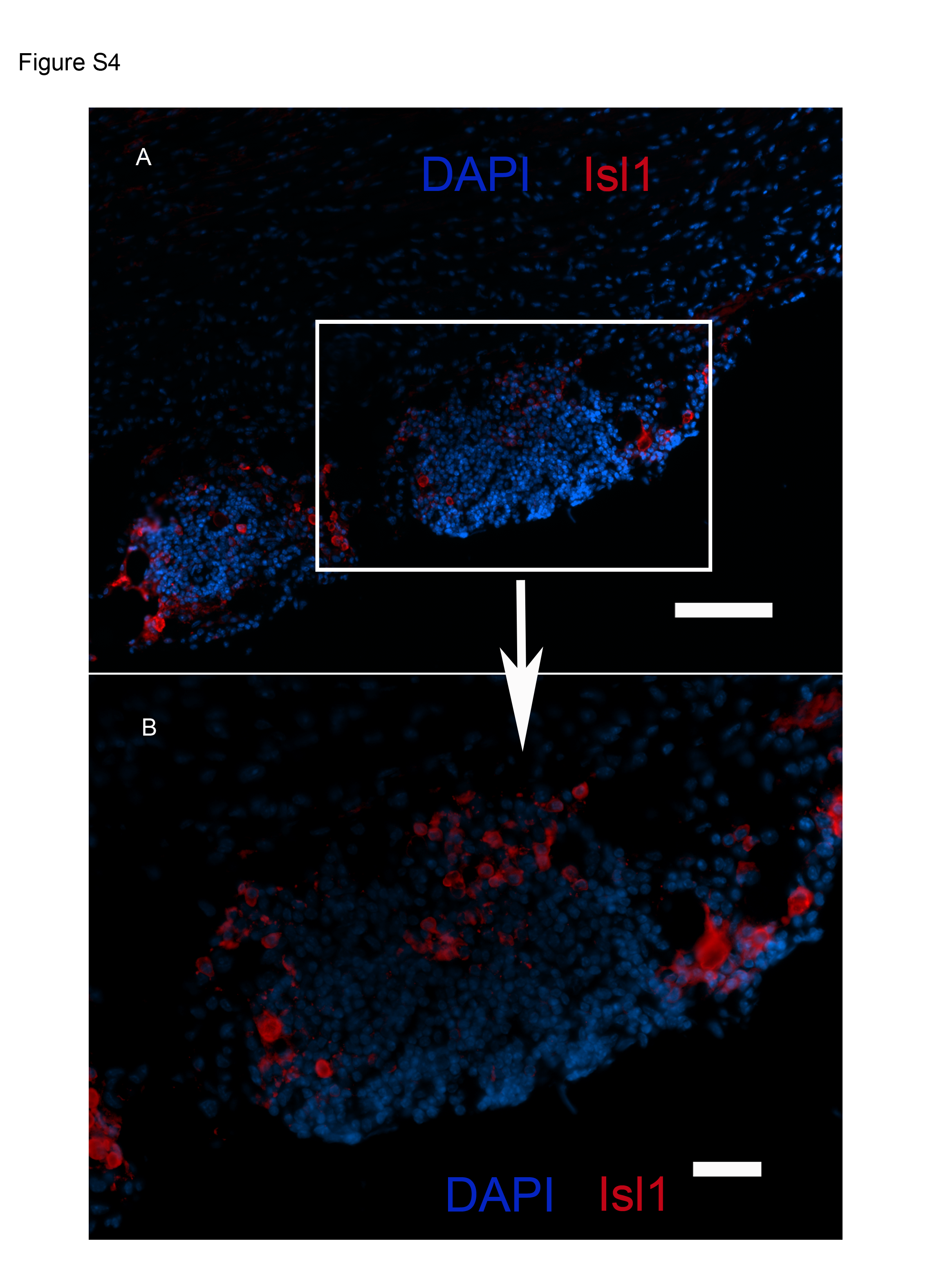

Supplement: Figure S4 — Isl1+ cells in post-MI heart. After 7 days post-MI, Isl1+ cells were detected in epicardium at the border of infarct region by immunohistochemical staining in 9 month old mice at lower power (scale bar = 35 µm) (A) and higher power (scale bar = 100 µm) (B) (N = 3). (TIF) [file pone.0030329.s004.tif]

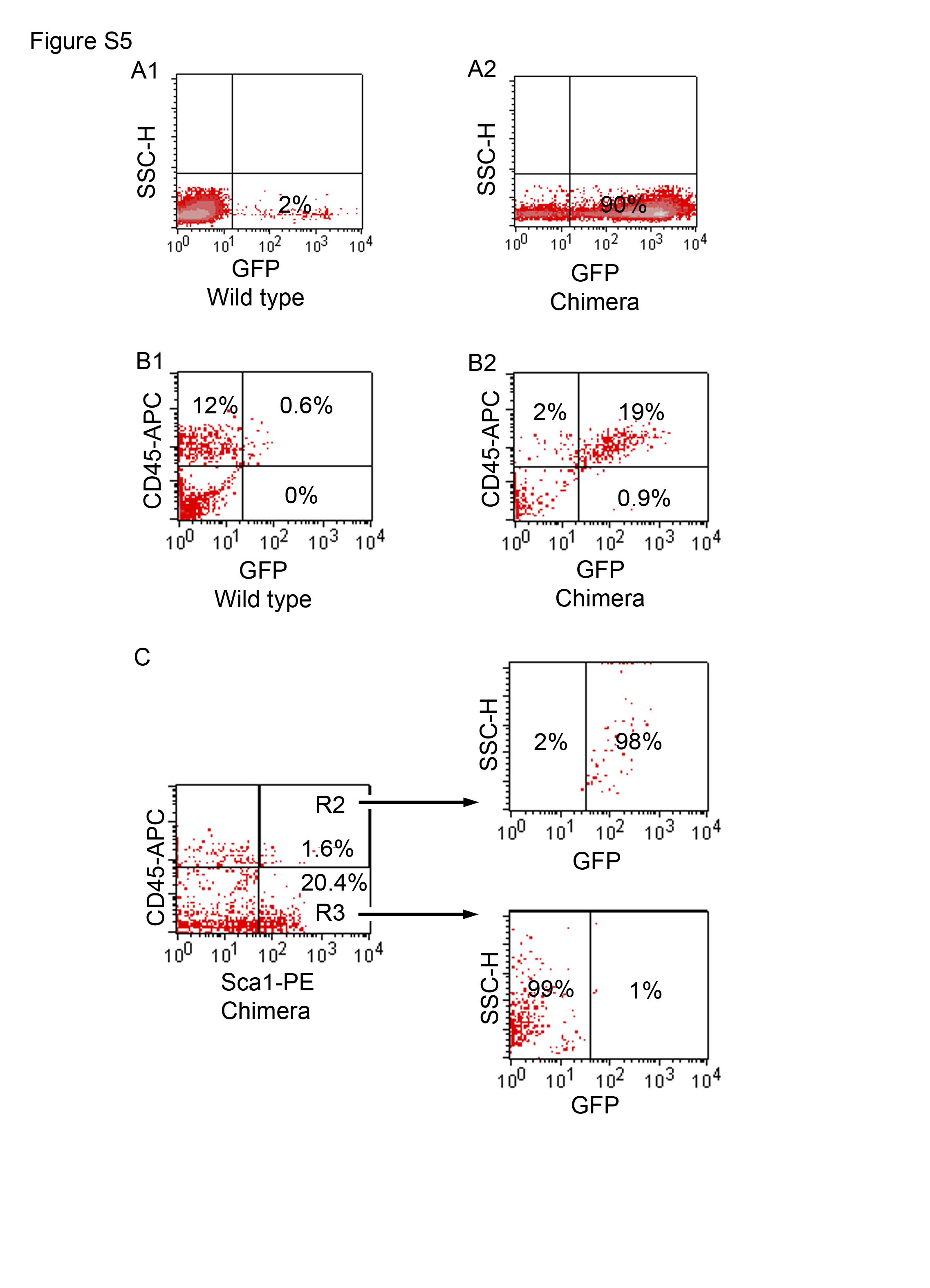

Supplement: Figure S5 — Sca-1+CD45- cells in CSs from chimeric mouse are GFP negative. Flow cytometric analysis GFP+ cells in peripheral blood mononuclear cells in wild type mouse (A1) and in chimeric mouse 5 months post-transplantation (A2). FACS showed CD45+ cells in CSs from wild type mouse (B1) and CD45+GFP+ cells in CSs from chimeric mouse (B2). FACS showed Sca-1+CD45- cells in CSs from chimeric mouse were GFP negative, whereas Sca-1+CD45+ cells were GFP positive (C). Typical results are shown (N = 4). (TIF) [file pone.0030329.s005.tif]

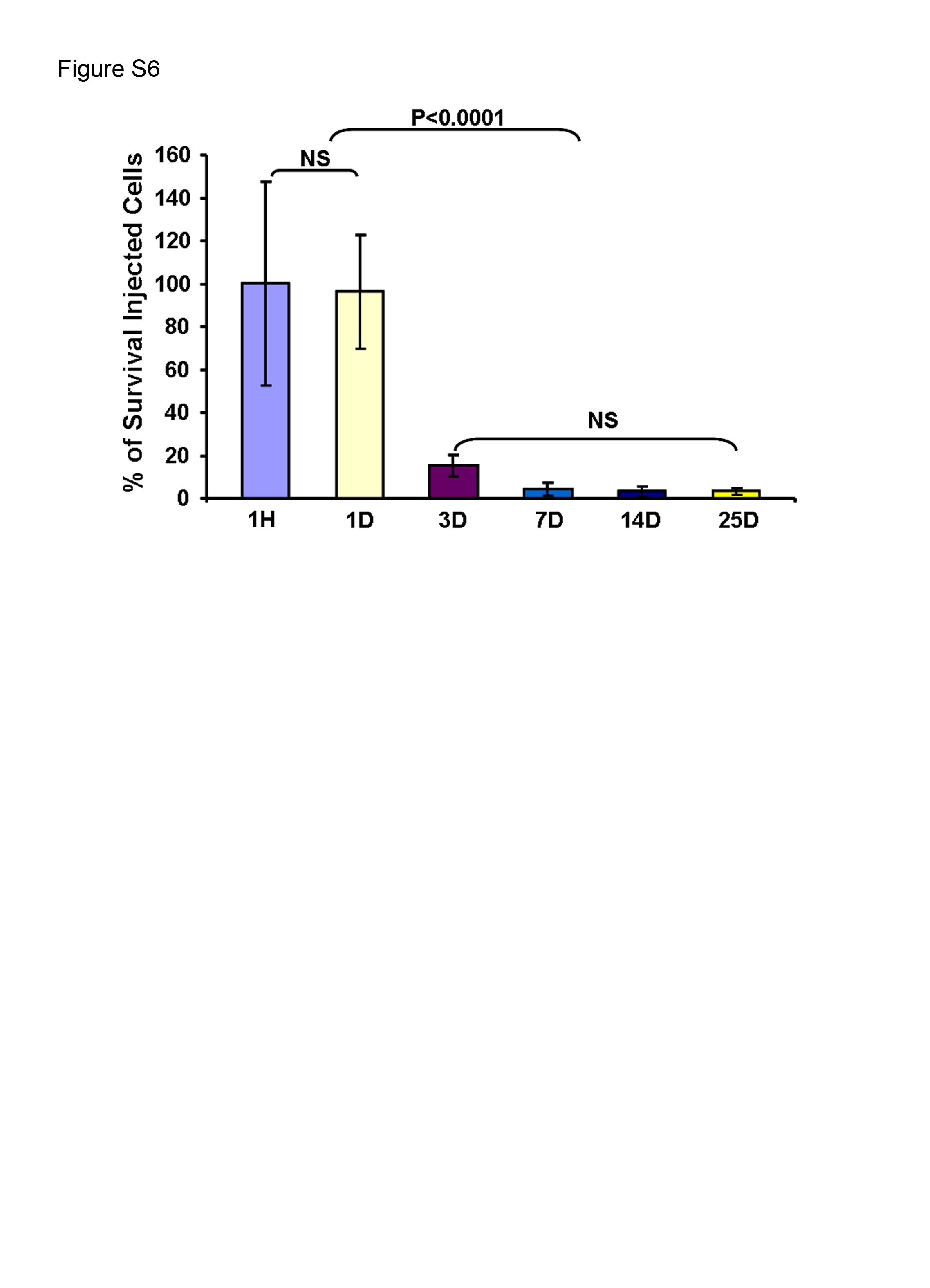

Supplement: Figure S6 — The level of engraftment and persistence of injected cells in infarcted hearts. Real-time RT-PCR (Taqman) was used to compare GFP expression in hearts injected with cloned Sca-1+CD45-GFP+ cells. The injected hearts were harvested at 1 hour and 1, 3, 7, 14 and 25 days post-injection. Results show as GFP mRNA expression relative to histone 3.3A. The expression level of GFP in the heart collected 1 hour post-injection was used to represent 100% of injected cells. H: hour; D: day. Typical results are shown (N = 3). (TIF) [file pone.0030329.s006.tif]

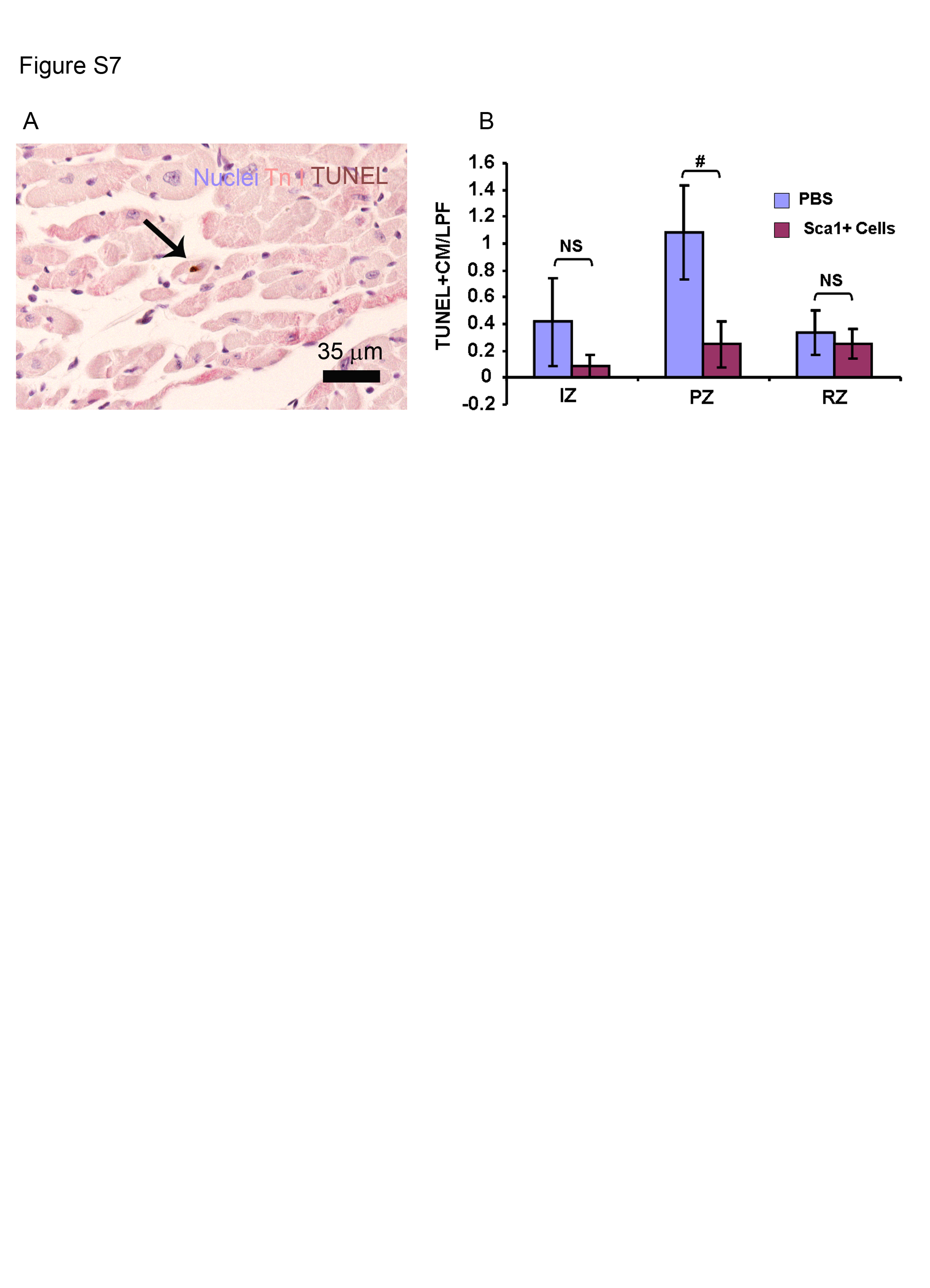

Supplement: Figure S7 — Injected Sca-1+CD45- cells reduce cardiomyocyte apoptosis. Typical image showed TUNEL+/Troponin I+ cells (black arrow) (A). Sca-1+ cell injection resulted in a significant reduction of TUNEL+/Troponin I+ cells in the peri-infarct zone (PZ), but not in the infarct zone (IZ) and remote zone (RZ) compared to the control group 25 days post-injection (B). TnI, troponin I; TUNEL, terminal deoxynucleotidyl transferase dUTP nick end labeling; CM, cardiomyocyte; LPF, low power field (20 x magnification); Data are shown as mean±SEM (N = 6). #<0.05. (TIF) [file pone.0030329.s007.tif]
